# Origins, Impacts, and Mitigation Strategies of Strain in Efficient and Stable Perovskite Solar Cells

**DOI:** 10.1002/smsc.202300014

**Published:** 2023-04-24

**Authors:** Xuxia Shai, Weitao Chen, Jiale Sun, Jinghui Liu, Jiangzhao Chen

**Affiliations:** ^1^ Institute of Physical and Engineering Science/Faculty of Science Kunming University of Science and Technology Kunming 650500 China; ^2^ Key Laboratory of Optoelectronic Technology & Systems (Ministry of Education) College of Optoelectronic Engineering Chongqing University Chongqing 400044 China

**Keywords:** metal halide perovskites, perovskite solar cells, strain, strain engineering, strain regulation

## Abstract

Because of their straightforward manufacture and exceptional photovoltaic efficiency, perovskite solar cells (PSCs) have quickly become appealing rivals in the photovoltaic sector. The residual strain, which prevents their practical use, keeps the efficiency and long‐term stability below practical bounds. Herein, the causes of strain in metal‐halide perovskites that are pertinent to photovoltaic applications, as well as how strain affects the materials’ physical characteristics and solar performance and how to control it, are discussed. Finally, a perspective on future strain engineering to support robust and effective PSCs is proposed. This review provides a comprehensive understanding of strain from origins, effects, to regulation, which would enhance the research enthusiasm on strain engineering to drive further improvements in performance especially stability of PSCs toward commercialization.

## Graphical abstract

Strain in metal halide perovskites and perovskite solar cells (PSCs) is increasingly gaining attention from the research community. This review provides an extensive understanding of strain from origins, effects, to regulations, which would drive further improvements in efficiencies and especially stabilities for PSCs toward commercialization.

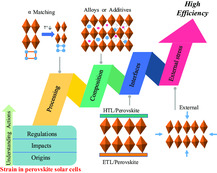

## 1 Introduction

Lead halide perovskites have captured the interest of a significant number of researchers working in the field of solar applications for more than 10 years. This is likely due to the extraordinary optoelectronic capabilities of these materials.^[^ ^1^, ^2^, ^3^ ^]^ As a consequence of systematic optimization, which mainly concentrates on perovskite composition engineering, solvent engineering, and interface engineering, the photoelectric conversion efficiency (PCE) of perovskite solar cells (PSCs) has experienced unprecedented growth.^[^ ^4^ ^]^ The versatility of perovskite materials and diversity of the corresponding device architectures express a tempting vista of commercialization, but the mechanically soft nature leads them trending to structural strain, resulting in significant vulnerability to moisture, air, heat, or prolonged exposure to sunlight that hinders their practical application.^[^ ^5^, ^6^, ^7^ ^]^

In semiconductors, strain is a fictitious physical term that may be modulated to alter their optoelectronic capabilities,^[^ ^8^, ^9^ ^]^ and the strain in perovskite films may be produced both internally and externally. Perovskite crystals experience intrinsic strain, which without any external stress results from the nonsymmetry of crystal or distortion of crystal lattice periodicity. In contrast, perovskite crystals have been shown to exhibit an extrinsic external strain. This kind of strain may be characterized by the nonperiodic behavior of the crystal lattice in the presence of external stimuli. There is widespread consensus that strain significantly degrades the quality of perovskite thin films and that the structural disorder at specific crystallographic positions is the primary factor that contributes to the diffuse phase transition. In order to design efficient strain engineering solutions, it is crucial to have a complete understanding of the causes and effects of strain.

**Figure 1:**
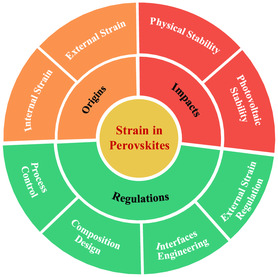
Outline of the origins, impacts, and mitigation strategies of strains in perovskite films and PSCs.

Currently, strain engineering in PSCs has been widely adopted to increase photovoltaic performance.^[^ ^10^, ^11^, ^12^, ^13^ ^]^ Various measures to modulate strain have been frequently attempted, including using high‐pressure nitrogen extraction,^[^ ^14^ ^]^ light‐induced lattice expansion,^[^ ^15^ ^]^ component regulation,^[^ ^16^ ^]^ interface modification,^[^ ^17^, ^18^ ^]^ and so on. In view of the great contributions of strain engineering to photovoltaic performance, a timely and comprehensive review is urgently needed to advance the development of perovskite photovoltaic. In this review, the causes of strain as well as its effects on perovskite films and PSCs are investigated in a methodical manner and explored at length. Subsequently, various strategies to control the strain are summarized (**Figure** **1**). Finally, we propose a perspective for future development direction on strain engineering.

## 2 Origins of Strain

Numerous variables may lead to strain, including the composition of precursor solutions, the substrate's mismatched thermal expansion coefficient (*α*), and the residual strain of perovskite films which is mostly determined by the development and measurement conditions, as well as by external stimulation. The creation of defects like grain boundaries (GBs) or dislocations as well as an imbalance in the thermal expansion coefficient are important driving mechanisms for these stresses. In this regard, the various affecting factors resulting in strain or instability from internal and external during the experiments are discussed in the following paragraphs, which is beneficial to understanding the origins of strain.

### 2.1 Internal Strain

Because of the soft ionic character of perovskite materials, improper formation of perovskite crystals often occurs even in the absence of any external force effects. This leads to the distortion of crystal symmetry or crystal lattice periodicity, which is the primary cause of internal strain. According to the crystal structure characteristically, octahedra tilting, complicated crystallization, and heterogeneous phase should mainly blame.

#### 2.1.1 Octahedra Tilting

Corner sharing of metal halide [BX_6_]^4−^ octahedra produces the classic 3D perovskite crystal structure, with A‐site cations filling the octahedral voids. This structure corresponds to the chemical formula ABX_3_. There are several ABX_3_ formulations available now as a result of more than 10 years of intensive study in the area of halide perovskites. But the variety of materials that are readily accessible is severely constrained because of the requirements for an acceptable bandgap, long‐term stability, and minimal nonradiative recombination. FA^+^, MA^+^, and Cs^+^ together make up the majority of the A‐site cations, and the leading position of lead in the B position must be ensured in order to accomplish effective photovoltaic conversion. In order to increase the photoelectric characteristics, the optimum narrow bandgap requires that the X‐site anions mostly consist of I, with just a little amount of Br or Cl doping.^[^ ^19^ ^]^ The stability of the A, B, and X components, as well as the bonding properties between them, has a significant impact on ion migration, lattice strain, and phase change of perovskites that occur during the operation of perovskite devices. Especially, the [PbX_6_]^4−^ octahedral structure functions as both a framing for perovskites and a transmission channel for photogenerated carriers, determining the performance of perovskite materials intimately.^[^ ^20^ ^]^

Organic cations and [PbX_6_]^4−^octahedra, two structural features of lead halide perovskites, serve as structural stabilizers and supporting skeleton, respectively.^[^ ^2^ ^]^ The A cation was generally not thought to directly influence the band‐edge structure of perovskite since the orbital states of the B and X species make up the bulk of it. This led to the assumption that the A cation had no effect on the optoelectronic properties of organic–inorganic lead halide perovskites.^[^ ^21^ ^]^ The [BX_6_]^4−^ octahedron connected by angle sharing forms the basic framework of the material, determines the lattice parameters of perovskite, and is closely related to the crystal structure change by inflation, contraction or tilting, etc. Unfortunately, the corner‐sharing octahedral skeleton's poor bonding strength causes a drop in activation energy (*E*_a_) and the creation of defects, which speed up ion migration and cause the lattice structure to collapse.^[^ ^22^ ^]^ In practice, because of the mismatched *α* between the substrate and perovskite materials, it is straightforward to add tensile strain during the cooling phase. This causes the perovskite's lattice to expand under tensile strain, which directly influences the octahedral structure by changing the bond angle of B—X—B and B—X bond length. More seriously, if [BX_6_]^4−^ octahedra undergo a high twist, some B—X bonds will break and cause a nonperovskite structure unexpectedly.

**Figure 2:**
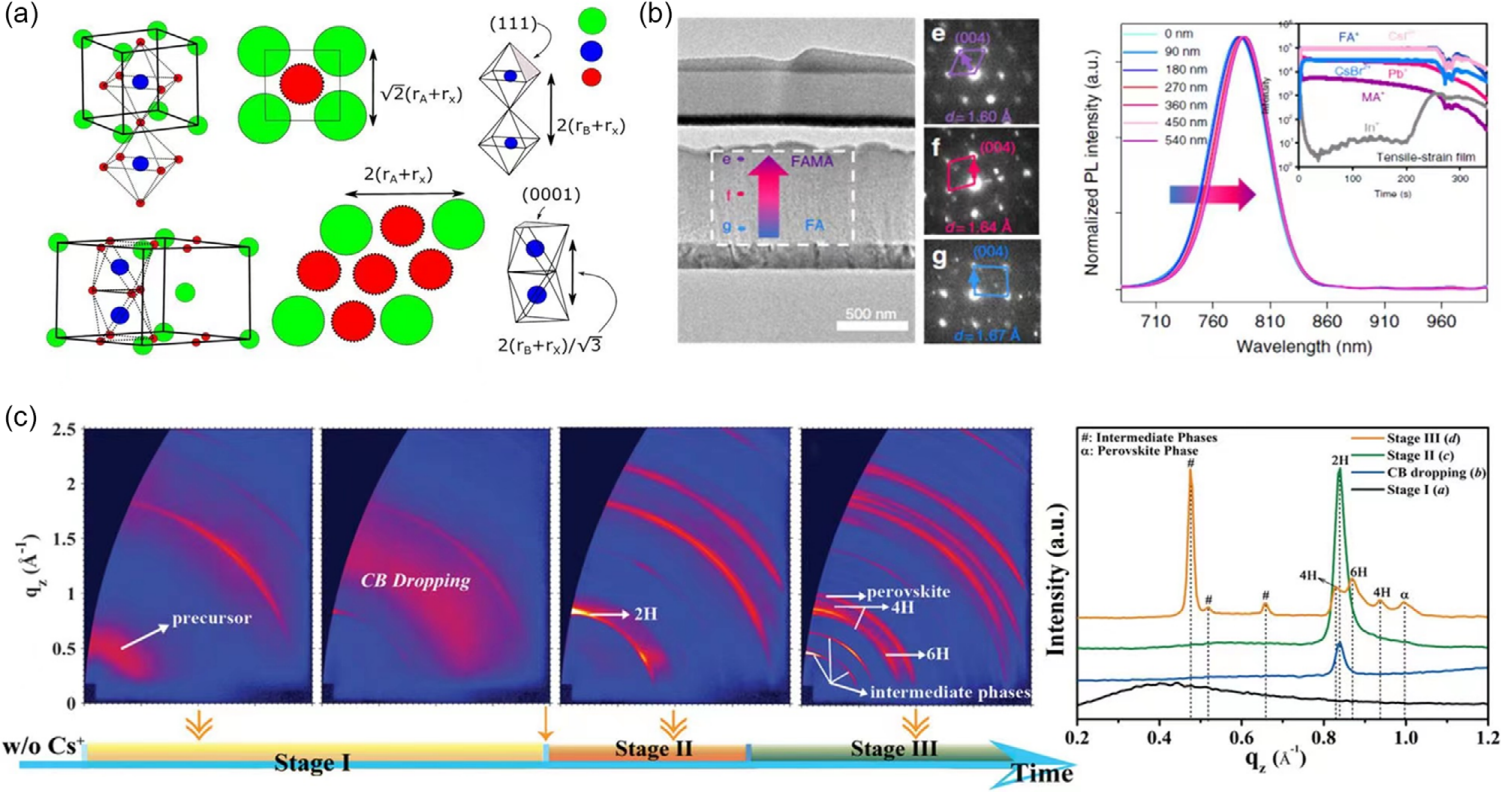
a) Schematic diagram of the strain formation process of FAPbI_3_ perovskite, during the structure transformation from pseudocubic α‐phase to hexagonal δ‐phase. Reproduced with permission.^[^ ^24^ ^]^ Copyright 2016, American Chemical Society. b) The cross‐sectional transmission electron microscopy image of (FAPbI_3_)_0.85_(MAPbBr_3_)_0.15_ PSC, and e,f,g) respectively corresponding to the nanobeam electron diffraction patterns of points e, f, and g in the perovskite layer, indicating a change in phase structure and domains with various lattice parameters from the perovskite film's surface to its bottom. Additionally, the inset shows the equivalent time‐of‐flight secondary‐ion mass spectrometry depth profiles with tensile strain for the PL depth profile of the confocal fluorescence microscopy. Reproduced with permission.^[^ ^40^ ^]^ Copyright 2019, Springer Nature. c) The 2D grazing‐incidence wide‐angle X‐ray scattering (GIWAXS) patterns at different crystallization stages for FA_0.83_MA_0.17_Pb(I_0.8_Br_0.2_)_3_ film and the corresponding intensity of the *q*_*z*_ direction, indicating a complicated crystallization process within. Reproduced with permission.^[^ ^43^ ^]^ Copyright 2019, Wiley‐VCH.

Additionally, the physiochemical properties and stability of the perovskites have also been demonstrated to be significantly influenced by the A cation, either intrinsically or extrinsically.^[^ ^23^ ^]^ Internal strain in the perovskite lattice is relevant to the mismatch in atomic size. Structural variety that results in markedly different optical and electrical properties is encouraged by the tilting of the [PbI_6_]^4−^ octahedra brought on by the ionic size mismatch between the A‐site cation and the lead halide cage. For instance, the FA^+^ cation's higher size is responsible for the [PbX_6_]^4−^ octahedron's local tilting, which causes the Pb—X—Pb bond angle to deviate from the ideal 180° and causes local lattice distortion. In detail, the pure *α*‐FAPbI_3_ has a sixfold corner‐sharing octahedral perovskite structure (**Figure** **2**a), while the larger size of FA^+^ molecule (A‐sites, FA^+^ = 253 pm) than that of Pb^2+^ (B‐sites, Pb^2+^=120 pm) sits in 12‐fold coordination, in order to adapt the relatively large FA^+^ to relieve the strain; it transforms into the face‐sharing structure (*δ* phase) to lower the total energy of the system. We shrink the distance between smaller atoms (Pb–Pb) to achieve the separation between bigger atoms; the FA–FA shorter distance of hexagonal structure is $\sqrt{2}$ times bigger than that of *α*‐phase.^[^ ^24^ ^]^

Even though these steric effects dominate the octahedral tilting, it is thought that the hydrogen bonding between [BX_6_]^4−^ octahedra and A^+^ cations is what causes the octahedral tilt in hybrid halide perovskites also.^[^ ^25^ ^]^ Because of the weak secondary hydrogen bonding (with bonding energies less than 0.1 eV per bond) contact between A^+^ cations and [BX_6_]^4−^ octahedra, the reorientation of organic cations may be thermally activated at a certain temperature.^[^ ^26^ ^]^ The activated reorientation of the organic A cation resulting from off‐centering or heterogeneous distribution of A cations leads to [PbI_6_]^4−^ octahedra tilting, continuous change in lattice parameters, and local symmetry‐broken state, causing the largely heterogeneous strain across the perovskite films.^[^ ^27^ ^]^ It should be noted that the order–disorder transition of the organic cations has a substantial impact on how the inorganic octahedra are aligned. For example, the real‐time neutron diffraction studies on MAPbBr_3_ perovskites suggested that order‐to‐disorder transformation temperature of MA^+^ cations is between 100 and 153 K, the disordered MA^+^ has great influence on inorganic octahedrons and leading tilt or even twist of them, resulting in the phase change from orthorhombic to tetragonal, and the structural change from multidomain to single‐domain structure.^[^ ^28^ ^]^

#### 2.1.2 Complicated Crystallization and Heterogeneous Phase

Multiple‐ion composite perovskites have drawn a lot of interest due to their large range of adjustable bandgaps (*E*_g_ = 1.18–2.3 eV).^[^ ^29^, ^30^ ^]^ Several compositions have shown excellent photovoltaic performance, such as (Cs/MA/FA)Pb(I_*x*_Br_1−*x*_)_3_, MA_1−*y*_FA_*y*_Pb(I_*x*_Br_1−*x*_)_3_, and MASn_*x*_Pb_1−*x*_I_3_.^[^ ^30^, ^31^, ^32^ ^]^ However, due to the complicated crystallization process from the changeable composition, high‐quality perovskite films with low defect density and excellent crystallinity are still difficult to manufacture.^[^ ^33^, ^34^, ^35^, ^36^, ^37^, ^38^ ^]^ It is a consequence from the multiple precursors in ABX_3_ construction, leading a detrimental effect on the characteristics of perovskite films and reducing device performance.^[^ ^39^ ^]^ Furthermore, the size differences between the halide anions and cations, in particular in mixed‐cation perovskites (MCPs) and mixed‐halide perovskites (MHPs), would cause a decline in the stability of twisted perovskite crystals during the further crystallization processes.^[^ ^2^ ^]^ Therefore, it is easy to find the inhomogeneous distribution of elements in multiple component perovskites, resulting in perovskite domains with distinct lattice parameters inducing extra strain with the film (Figure 2b).^[^ ^40^ ^]^ However, MHPs and MCPs are essential parts of high‐performance PSCs, and the highest‐efficiency PSCs are based on MCPs.

To further understand the crystal growth situation, in situ testing technologies have been widely used to conduct research on perovskite crystallization kinetics as well as strategies for changing perovskite crystallization kinetics.^[^ ^41^ ^]^ Pistor et al. found that the phase formation of MAPbI_*x*_Cl_3−*x*_ is significantly influenced by the ratio of the MAI to PbX_2_ flux, and the MAI to PbI_2_ flux ratio was found to significantly influence the orientation of the deposited films. Upon annealing, the decompositions of MAPbI_*x*_Cl_3−*x*_ were detected with temperatures above 200 °C, accompanied by the decreased peak intensities and increased full‐width‐half‐maximum of the peaks of the perovskite (100), (200), and (210), and PbI_2_ and Pb reflexes appeared in the X‐ray diffraction (XRD) system, which exemplifies the intricate crystallization procedures of MHPs.^[^ ^42^ ^]^ In addition, it was found that the mixed perovskites (FA_0.83_MA_0.17_Pb(I_0.8_Br_0.2_)_3_) produced by the spin‐coating process is involved in three complex stages: 1) precursor solution, 2) hexagonal *δ*‐phase, and 3) intermediate phases and perovskite *α*‐phase.^[^ ^43^ ^]^ Within the annealing window, thermal annealing manipulation is carried out (which extends throughout stage 2), and high‐quality mixed‐perovskite films (relating cells with good photovoltaic performance) were made by controlled storage (Figure 2c).

Although the mixed‐cation strategy is also accompanied by intricate crystallization kinetics, the MCPs are still often employed to build high‐performance perovskite photovoltaic equipment. It must be noted that the strain and grain orientation spread significantly vary depending on the crystallization kinetics in the perovskite growth process, which results in disparate crystal properties and eventually influences the film quality. So, it is urgent to investigate the complicated crystallization properties of the perovskite film, and regulation strategy on crystallization kinetics must be further explored.

### 2.2 External Strain

Different from the internal strain of the perovskite crystal discussed earlier, the nonperiodic behavior of the crystal lattice will occur in the presence of external stimuli. The following two aspects are the main causes of external strain, one is the mismatched lattice and thermal expansion coefficient (*α*) and another is the external stress such as illumination, external pressure, temperature and electrical bias etc. Below are the specific impacts.

#### 2.2.1 Lattice and Thermal Expansion Coefficient (α*) Mismatch*

Investigating the reason for external strain, the incompatibility *α* between adjacent layers bears the brunt undoubtedly. Strains in perovskite caused by the mismatched *α* between adjacent layers (such as ITO, FTO, or glass substrate and electron‐ or hole‐transport layer) are frequently generated in PSCs.

**Figure 3:**
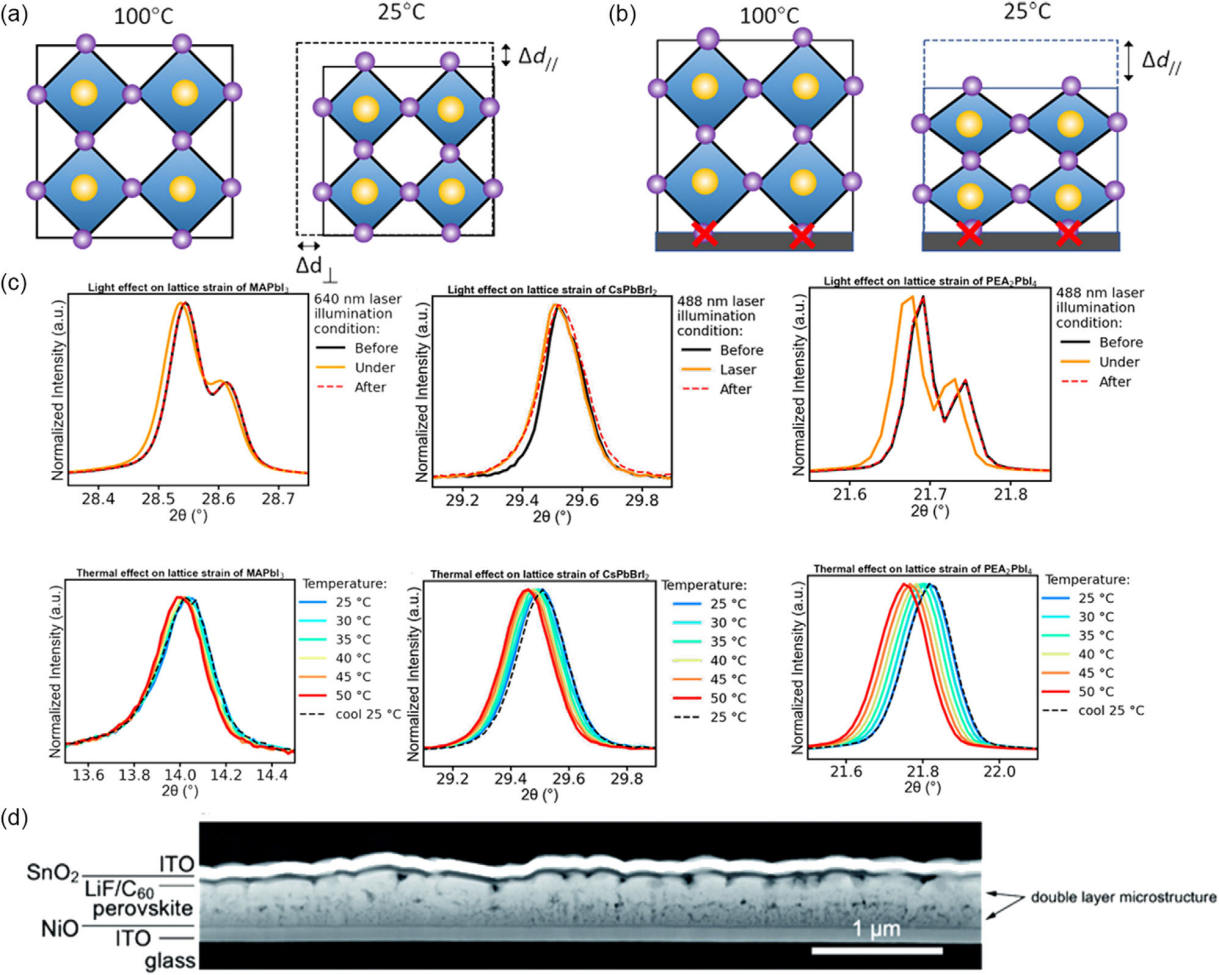
a,b) The perovskite crystal with (a) and without (b) substrate during cooling, revealing the strain formation process due to the adhesion of substrate. a,b) Reproduced with permission.^[^ ^44^ ^]^ Copyright 2017, American Association for the Advancement of Science. c) The XRD results of various cations perovskite (MAPbI_3_, CsPbI_3_, and CsPbBrI_2_) under external conditions of light and thermal. Reproduced with permission.^[^ ^56^ ^]^ Copyright 2021, American Chemical Society. d) The scanning transmission electron microscopy (STEM) of solar cell under reverse bias at −5 V, the degradation corresponding to the large voids and a double‐layer structure. Reproduced with permission.^[^ ^58^ ^]^ Copyright 2020, Royal Society of Chemistry.

Huang et al. reported that the quality and intrinsic stability of perovskite films were significantly impacted by its large thermal expansion coefficient.^[^ ^44^ ^]^ They found that the average linear expansion coefficient of the tetragonal phase MAPbI_3_ was 6.1 × 10^−5^ K^−1^, which is more than one order of magnitude larger than that of the glass or ITO substrate (*α*_glass_ = 0.37 × 10^−5^ K^−1^ and *α*_ITO_ = 0.85 × 10^−5^ K^−1^).^[^ ^45^ ^]^ When ignoring the incompatibility *α* between the substrates and perovskite films, the positive thermal expansion coefficient caused it to tend to contract upon cooling to ambient temperature, potentially resulting in an indistinguishable contraction in both out of plane and in plane. In other words, the contracts of the crystal planes spacing perpendicular to the substrate (Δ*d*_∥_) can be equivalent to that parallel to the substrate (Δ*d*_⊥_), if the thermal expansion coefficient is equivalent between the substrate and the perovskite (**Figure** **3**a). But because of the substrate's adhesion and the mismatched *α* between the perovskite layer and substrate, the perovskite in touch with the latter is unable to compress, leading to tensile strain in the in‐plane direction. The film also shrinks more in the out‐of‐plane direction, as a result of cooling, which makes up for the reduced lateral shrinkage by increasing the compressive strain in this direction (Figure 3b). In addition, residual strain in perovskite is closely associated with the substrate, and the existence of a temperature gradient across the perovskite layer may also lead to an unusual volume shrinkage during cooling and a gradient in strain inside the film. For instance, with a larger thermal expansion coefficient of *α* = 2 × 10^−5^ to 8 × 10^−5^ K^−1^ of poly(ethylene terephthalate) (PET), the MAPbI_3_ film on it has a significantly smaller lattice strain than that on the ITO/glass substrate.^[^ ^12^ ^]^ Furthermore, different from the aforementioned mismatched *α*, the substrate and perovskite's unique crystal plane spacing may also lead to a mismatch in the lattice, which can induce compressive or tensile strain.^[^ ^13^ ^]^ Significantly, due to the perovskite's intense adherence to the substrate, it is difficult to entirely remove strain in perovskite films created during high‐temperature processing.

Generally, thermal annealing is advantageous for grain development and coarsening, increasing the carrier transport characteristics, and lowering the density of GB defects, leading the thermal‐introduced strain of the substrate‐grown perovskite films inevitably. Therefore, to minimize the fabrication‐induced strain and enhance perovskite's intrinsic stability, low‐temperature formation or matched selection of thermal expansion coefficient substrates should be taken into account. However, an annealing process is required for perovskite crystallization and phase structure change, and as a result, the thin films are always subjected to very high thermal stress.^[^ ^44^ ^]^ In addition, a residual interfacial strain would also emerge from the mismatched lattice constants of two neighboring semiconductors, which must be taken into account during the manufacturing of the device, even ignoring the previously described incongruent thermal growth of the substrates and perovskite layers. As a result, the interface between perovskite and an electron or hole transport layer is where the external strain initially manifested itself during PSC development. In contrast to the previously described temperature coefficient mismatch, the residual stresses persist even if the perovskite crystallizes at ambient temperature.

#### 2.2.2 External Stress Conditions

Notably, the external conditions including illumination, external pressure, temperature, electrical bias, etc. are usually exerted during the operation of a photovoltaic device, inducing the additional strain in perovskite unavoidably.^[^ ^46^, ^47^ ^]^ Under incident light, a number of strain‐related variables have been established, such as photostriction,^[^ ^48^, ^49^, ^50^ ^]^ photothermal effect,^[^ ^51^, ^52^, ^53^ ^]^ and build‐up of electric field.^[^ ^54^ ^]^ The structural dynamics of perovskite films are significantly influenced by light‐induced strain, which also has an impact on the stability and optoelectronic characteristics of perovskite‐based devices. In MAPbI_3_, Wang et al. noted a massive 41 200 p.p.m. photostrictive response (i.e., light‐induced lattice change). It has been proposed that the hydrogen bond between N and I may become weaker due to photogenerated carriers, which would then cause the strong photon‐lattice coupling (lattice dilation).^[^ ^48^, ^55^ ^]^ In particular, in MHPs, light‐induced strain is significantly influenced by the A‐site cation.^[^ ^56^ ^]^ As was usually the case of thermal‐induced or light‐induced lattice expansion in single‐cation perovskite such as MAPbI_3_, CsPbBrI_2_, and PEA_2_PbI_4_, a diffraction shift to the left was found after changing conditions (Figure 3c), and the shift degree was closely related to A cation. Huang et al. demonstrated that the photothermal‐induced (an increase in device temperature) expansion is primarily accountable for the light‐induced strain (heating effect induced by light exposure expansion) in MAPbI_3_. The excess charge carriers and elevated temperature lead to the formation of additional recombination centers, ultimately facilitating the light‐induced degradation.^[^ ^52^ ^]^ The tensile strain from thermal expansion during light soaking, which was demonstrated to result in the distorted inorganic [PbX_6_]^4−^ framework with less strongly tilted octahedra due to longer and weaker Pb—X bonds, resulted in a lower activation energy for ion migration and a lower formation energy for defects.^[^ ^55^ ^]^ Therefore, the strains resulting from photothermal‐induced lattice expansion can speed up the degradation of perovskite. On the other hand, different from the above disadvantages of light‐induced strain with lattice expansion in single‐cation perovskite, according to Tsai et al.,^[^ ^50^ ^]^ the uniform lattice expansion that results from continuous illumination of the FA_0.7_MA_0.25_Cs_0.05_PbI_3_ film, which is attributed to strain relaxation under light, may likewise positively affect the FA film's optoelectronic properties.

A thorough investigation of the effects of bias and light on the structure of perovskite has been conducted by Baillie et al.^[^ ^47^ ^]^ Apart from the disorder of light‐induced strain, by applying positive bias voltages, a strong ferroelastic domain which is the source of an internal strain was observed, indicating an increase in structural disorder.^[^ ^57^ ^]^ While under the negative bias voltages, structurally domains are disappear, indicating the dominant migration of organic cations which result in surface damage. Research on the degradation mechanisms of PSC under reverse bias, revealing a phase segregation that was accompanied by the microstructure changes (Figure 3d), which begins when the cell is passed by the current.^[^ ^58^ ^]^ The reason for deterioration under reverse bias has been implicated in a variety of ways, which is related to charged species’ bias‐induced migration, severely limiting the photoelectricity stability and photoelectricity performance.^[^ ^59^, ^60^ ^]^

It is feasible to manipulate the optoelectronic properties and structural of perovskite by applying appropriate hydrostatic pressure, leading to narrowing bandgap and broadening the absorption spectrum that is beneficial for achieving better photovoltaic performance.^[^ ^61^, ^62^, ^63^ ^]^ However, because to its inherent lattice anisotropy, the perovskite lattice's bond lengths and angles may be changed by pressure engineering, leading to tensile or compressive strain.^[^ ^64^ ^]^ Tensile strain is thought to contribute to perovskite instability, by weakening bonding, increasing the growth of flaws, and decreasing the activation energy of ion migration; it hastens the dissolution of perovskites.^[^ ^65^ ^]^ Calculations based on relativistic first principles show that under compression strain, the octahedra are deformed by pressure, and as a result, the cation spins to fit into the cavity.^[^ ^66^ ^]^ Especially, under high pressure, perovskite films exhibit completely amorphized or irreversible phase transformations, which have diverse effects on the properties of perovskite, depending on its composition.^[^ ^67^ ^]^

In short, several external condition‐induced strains can affect the perovskite's properties; this might have an impact on how well the relevant optoelectronic devices work, either positively or negatively. Thus, the external strain must be precisely controlled and comprehensively evaluated during the operating conditions.

## 3 Impacts of Strain (Physical Stability and Photovoltaic Stability)

### 3.1 Physical Stability (Ion Migration and Phase Segregation)

**Figure 4:**
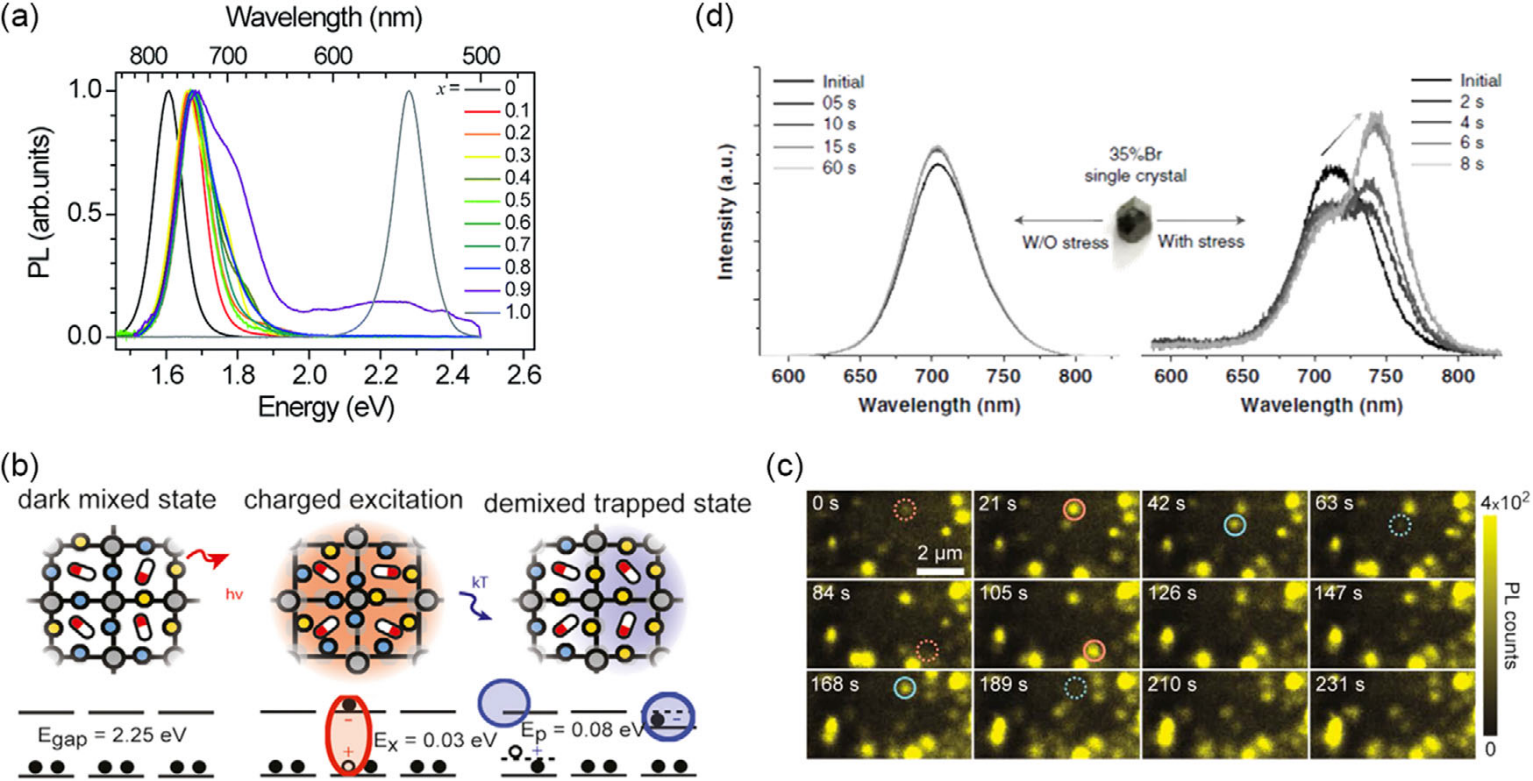
a) The normalized PL spectra of MAPb(Br_*x*_I_1−*x*_)_3_ films under light (457 nm, 10–100 mW cm^−2^) for 5–10 min. The new peak position emerging which is independent of composition and bandgap of perovskite. Reproduced with permission.^[^ ^74^ ^]^ Copyright 2015, Royal Society of Chemistry. b) Photoinduced polaron trapping related to phase separation, the blue and yellow spheres stand for Br^−^ ion and I^−^ ion, respectively, the pill shape is MA^+^, and the gray circles represent lead atoms. Reproduced with permission.^[^ ^71^ ^]^ Copyright 2017, American Chemical Society. c) Formation of iodide‐rich domains in MAPb(I_0.14_Br_0.86_)_3_ under low‐power laser (25 mJ cm^−2^) excitation, the iodide‐rich regions associate with the blinking cursor in the plate. The dotted and solid red circles represent the cluster appearing and upcoming locations, respectively; dotted and solid blue circles indicate the cluster position disappearing and about to disappear. Reproduced with permission.^[^ ^81^ ^]^ Copyright 2018, American Chemical Society. d) In situ PL tracking of MAPb(I_1−*x*_Br_*x*_)_3_ single crystals (for 35%Br) under illumination without (left) and with external uniaxial pressure (right). Reproduced under the terms of the CC‐BY Creative Commons Attribution 4.0 International license (https://creativecommons.org/licenses/by/4.0).^[^ ^83^ ^]^ Copyright 2020, The Authors, published by Springer Nature.

There are numerous effects of strain accumulation on lead halide perovskites’ stability.^[^ ^13^, ^44^, ^68^, ^69^, ^70^, ^71^, ^72^ ^]^ This section focuses on how ion migration is affected by tensile and compressive strain, promoting or preventing the occurrence of unfavorable perovskite phase segregation. The most active ions in the lattice for the organic inorganic hybrid lead halide perovskite are halide ions, due to much lower activation (*E*_a_) for halide ion migration than that of organic cation and lead ions (*E*_a_(Pb^2+^) >> *E*_a_(A^+^) > *E*_a_(X^−^)).^[^ ^54^, ^73^, ^74^ ^]^ Hoke et al. first reported the formation of halide‐enriched domains due to phase segregation resulting from halide migration,^[^ ^74^ ^]^ causing the original mixed composition's bandgap homogeneity to be broken, which leads to the creation of domains with lower and greater bandgaps. The photogenerated charges moved via phase segregation into iodide‐rich low‐bandgap domains, where they underwent recombination (**Figure** **4**a); it significantly affected how well the gadget performed. According to the data made thus far about the impact of strain on phase segregation, stress that is created at various length scales, from the atomic to the macrolevel, has a synergistic effect.^[^ ^72^ ^]^

The composition, strain, and crystal structure are all fundamentally related at the atomic level. For example, when MA^+^ or FA^+^ is substituted with Cs^+^, phase segregation is prevented by a reduction in the unit cell volume and the internal strain gradient brought on by the addition of the bigger organic cation to the perovskite structure. Additionally, the lattice's physical compression and the addition of Cs^+^ to lower the lattice parameter both have an equal influence on the phase separation process.^[^ ^75^, ^76^ ^]^ However, phase segregation is still present in Cs‐based mixed‐halide compositions,^[^ ^12^ ^]^ and triple‐cation (FA^+^/MA^+^/Cs^+^) compositions need longer time and greatly higher photon doses for halide redistribution than those that are required in MHPs with single or double cations.^[^ ^77^ ^]^ These changes in mixing ratio of cations lead to complex crystallization and defects formation, resulting in undesirable strain in perovskite films.^[^ ^72^ ^]^ In order to calculate the proper volume for a stable unit cell and comprehend the impact of differences in bond angles and length on the activation energy for halides’ migration, it is crucial to quantitatively quantify strain at the atomic level for mixed‐cation compositions.

According to the polaron‐based hypothesis, strain in the lattice is mostly caused by lattice polarization (polarons). In the perovskite lattice, polarons are produced together with the displacement of atoms when photogenerated charge carriers are present (Figure 4b).^[^ ^71^ ^]^ It was feasible to determine that charge carrier injection results in nanoscale structural distortions that are compatible with expansive‐type (i.e., tensile) strain using the femtosecond diffuse X‐ray scattering observations and modeling.^[^ ^78^ ^]^ Within few tens of picoseconds, polarons reach their maximum size and begin to decay following a transient excitation pulse that lasts for hundreds of picoseconds. The electromechanical characteristics of MHPs and their high ionic conductivities result in the formation of polaron populations, which provide a local strain gradient that promotes halide demixing and modifies the local free energy of halide mixing.^[^ ^79^ ^]^ The fact that the polaron clusters are heterogeneously created in both space and time provides further proof that the phase separation is a stochastic process that does not take place in an equilibrium state.^[^ ^80^ ^]^ For instance, the phase‐separated MAPb(I_0.14_Br_0.86_)_3_'s iodide‐rich areas were located using laser scanning excitation (Figure 4c).^[^ ^81^ ^]^ Polarons stabilize the I‐rich clusters and allow for stochastic nucleation, and the emergent locations of clusters are changed each time with the performation of reversible processes.

Mechanical deformation is a residual strain phenomenon that occurs on a large scale. It might be brought on by a number of different things, such as the perovskite crystallizing on a flexible substrate that has an incorrect coefficient of thermal expansion, the bending and twisting of thin films, or pressure from the outside. The stability of mixtures of mixed halides and the pace of phase segregation are both significantly impacted by strain on a macroscale, and the amount of strain that may be compensated for relies on the temperature of the operation as well as the electron transport layer (ETL) and hole transport layer (HTL) components of the perovskite.^[^ ^44^, ^82^ ^]^ Zhao et al.^[^ ^83^ ^]^ reported that the tensile stress (also known as mechanical deformation) applied in the in‐plane direction of MAPb(I_1−*x*_Br_*x*_)_3_ single crystals with a 35% bromide content and no phase segregation causes compression in the out‐of‐plane direction, which leads to the formation of domains rich in iodide (Figure 4d). They also explored the phase instability and the strain‐activated regime of single crystals with different contents of bromine, demonstrating that MAPb(I_1−*x*_Br_*x*_)_3_ with Br concentration less than 50% has strain activation on light‐induced halide segregation, while the phase segregation is an inherent process and strain only enhances it for Br concentration over 50%. Therefore, passivation strategies are only able to prevent phase segregation from occurring in compositions with strain‐activated phase segregation. In addition, the phase segregation is more obvious at the GBs for compositions that have strain‐activated phase segregation, but for compositions that have intrinsic phase segregation, the phase instability is visible both in the center and limit of grains. As the ion migration causes phase segregation, it is essential to inhibit phase segregation by suppressing ion migration to enhance device stability. Studies on transient pressure‐dependent absorption spectroscopy demonstrate that pressure has an impact on the kinetics and thermodynamics of the segregation of the MHP phase. It was discovered that under pressure, the phase segregation of certain mixed halides, such as MAPb(Br_*x*_I_1−*x*_)_3_ (*x* = 0.25, 0.5, and 0.7), greatly slows down. This is primarily because the effective activation energy of the halide migration process increases.^[^ ^55^ ^]^ Furthermore, Hutter et al.^[^ ^82^ ^]^ found that the compressing method either or applying external pressure, the mixed‐halide MAPb(I_1−*x*_Br_*x*_)_3_ perovskite stability against photoinduced halide segregation was greatly improved, which is due to the increased stability of the thermodynamics from the altered Gibbs free energy. From the above results, to protect against halide segregation and provide complete bandgap tunability of stable MHPs, it is hypothesized that any iodide:bromide ratio could be thermodynamically maintained by varying the crystal volume and compressibility. This would be possible, according to the theory. Furthermore, the HTL layer's external compressive strain compensation for tensile strain has the same result as physically compressing the perovskite film.^[^ ^12^ ^]^

In conclusion, strain has an impact on ion migration and thus causes a negative or positive effect on phase segregation of perovskites. It is a synergistic impact on the phase segregation that is brought about throughout a wide range of length scales, from the atomic level all the way up to the macrolevel.

### 3.2 Photovoltaic Stability (Carrier Dynamics and Defects)

It has been shown that strain may have a variety of consequences on how well perovskite films and the corresponding PSCs work, including effects on bandgap, defect characteristics, carrier transport, nonradiative recombination, and device carrier dynamics.^[^ ^64^, ^67^, ^68^, ^83^, ^84^ ^]^ It is predicted that the residual stresses, which are responsible for the bending of the energy bands in the perovskite absorber, would have some kind of effect on the carrier dynamics of the linked solar cell. Transport and extraction of the carrier across the interface can be improved by strain engineering.^[^ ^12^, ^13^, ^68^, ^69^, ^85^ ^]^

#### 3.2.1 Effect on Carrier Dynamics

**Figure 5:**
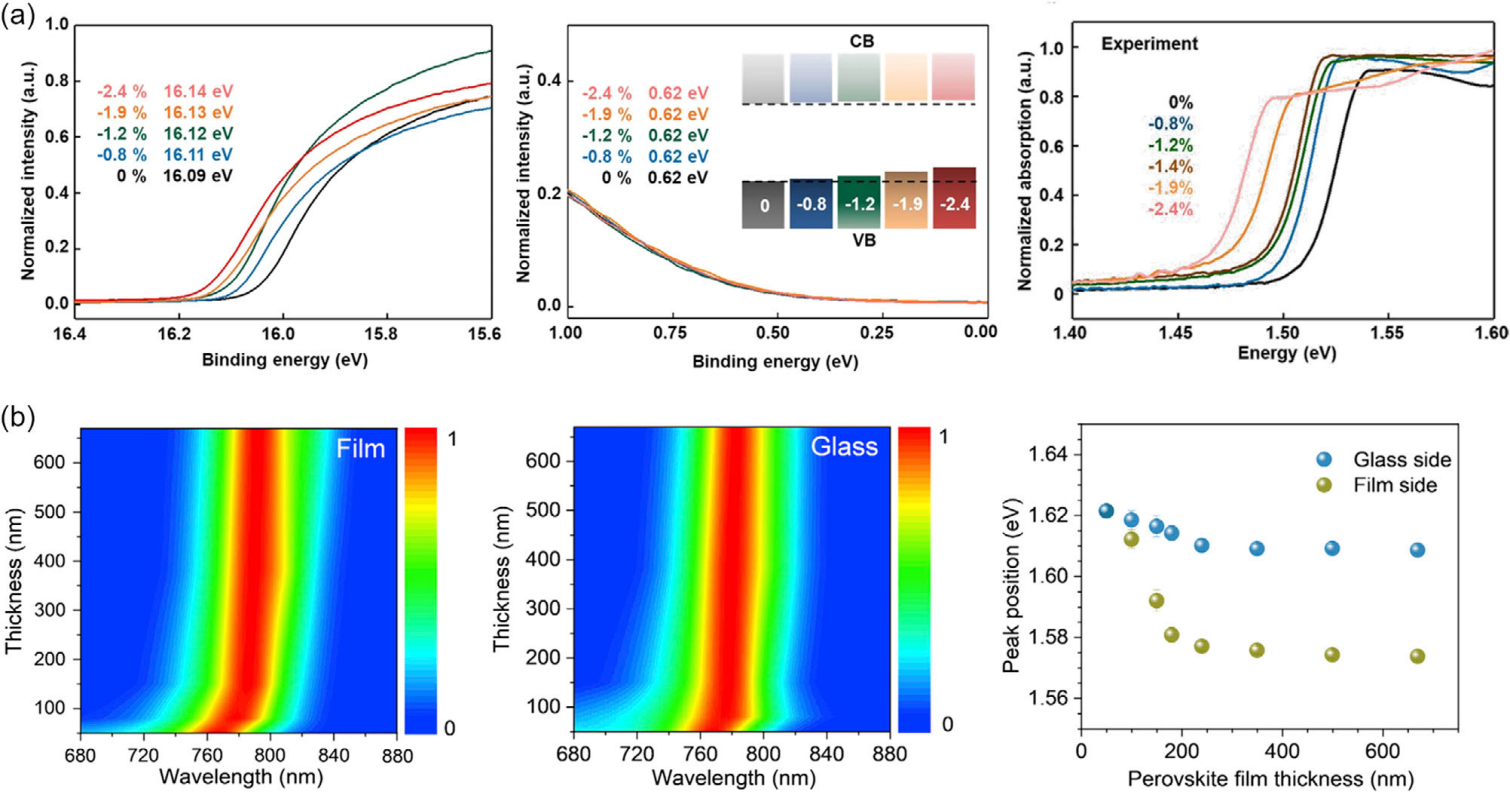
a) UPS spectra and absorption spectra of *α*‐FAPbI_3_ under different strains. Reproduced with permission.^[^ ^13^ ^]^ Copyright 2020, Springer Nature. b) The locations of samples’ thicknesses, which are differentiated by illumination from the film and glass sides, and the placement of the PL peak in relation to the thicknesses of perovskite films, are reflected in the normalized PL spectra. Reproduced with permission.^[^ ^88^ ^]^ Copyright 2022, Elsevier.

Xu et al. combined the experimental techniques with theoretical calculations to demonstrate that a compressive strain (less than or equal to −1.2%) on *α*‐FAPbI_3_ can effectively alter the structure of the crystal, increase the hole mobility of *α*‐FAPbI_3_, and reduce the bandgap.^[^ ^13^ ^]^ As can be observed in **Figure** **5**a's absorption spectra and ultraviolet photoelectron spectroscopy (UPS) of the strained *α*‐FAPbI_3_ thin films, the bandgap widens as a result of the absorption onset redshift as strain increases. Altering the hole‐related properties is the primary way in which the strain affects the overall carrier dynamics.^[^ ^85^ ^]^ Considering the Fermi‐level position and their energy difference with the valence band maximum (VBM), the VBM is brought higher than the conduction band minimum (CBM) with increasing strain. Because I 5*p* and Pb 6*s* orbitals make up the bulk of the VBM, the compressive strain increased cooperation among these orbitals, which resulted in an increase in the height of the VBM.^[^ ^86^ ^]^ Nevertheless, the deformation of the [PbI_6_]^4−^ octahedrons had less of an impact on the CBM, which was mostly made up of nonbonding localized states in Pb 6*p* orbitals.^[^ ^61^, ^87^ ^]^ This suggests that the in‐plane compressive strain enhances the VBM more than the CBM. The lattice deformation can adjust the structure of the electronic band and thus also the carrier dynamics. The strained (less than or equal to −1.2%) *α*‐FAPbI_3_ thin films obtained the enhanced hole mobility, but a higher level of strain will result in a higher density of dislocation that deteriorates the hole mobility.

At first glance, increasing compressive strain (relieving tensile strain) would enhance the stability of films. However, since the substrates were typically bent only in one direction, the applied stress was uniaxial. Due to Poisson's effect, it will produce opposing strains in directions that are perpendicular to the uniaxial strain. Therefore, it is simple to anticipate that the compressive and tensile stresses would occur along separate directions in the plane of the film. Additionally, as these features may be produced in such bending investigations and affect ion migration, the impact of wrinkles and fractures on the quality of crystal film cannot be disregarded. Furthermore, it was proved that the distribution of bandgaps within the perovskite bulk is graded due to subsistent strain. As shown in Figure 5b, with the increase in layer thickness, the PL was found to shift due to the strain caused by the perovskite bottom interface. Therefore, strain relaxation is crucial for improving the inherent stability of perovskite films, and it has been shown that strain‐free solar cells have improved stability and photovoltaic efficiency compared to strained devices.^[^ ^44^, ^88^ ^]^ Chen et al.^[^ ^40^ ^]^ found a good processing reproducibility with narrow distribution in PCEs of the strain‐free PSCs with the regular structure of ITO/SnO_2_/(FAPbI_3_)_0.85_(MAPbBr_3_)_0.15_/Spiro‐OMeTAD/Ag. The strain‐free devices demonstrated enhanced light harvesting efficiency over the whole absorption wavelength and improved fill factor (FF) and *V*_OC_ in comparison to the tensile strain devices.

#### 3.2.2 Effect on Defect Properties and Nonradiative Recombination

According to the report, strain‐induced structural flaws in polycrystalline perovskite films result in nonradiative recombination.^[^ ^89^, ^90^ ^]^ It has been shown that compressive strain improves perovskites’ intrinsic stability, increases the activation energy for ion migration, and increases the thermal and light stability of PSCs. On the contrary, tensile strain, weakening bonds, reducing the energy required to produce defects and to activate ion migration.^[^ ^12^ ^]^

**Figure 6:**
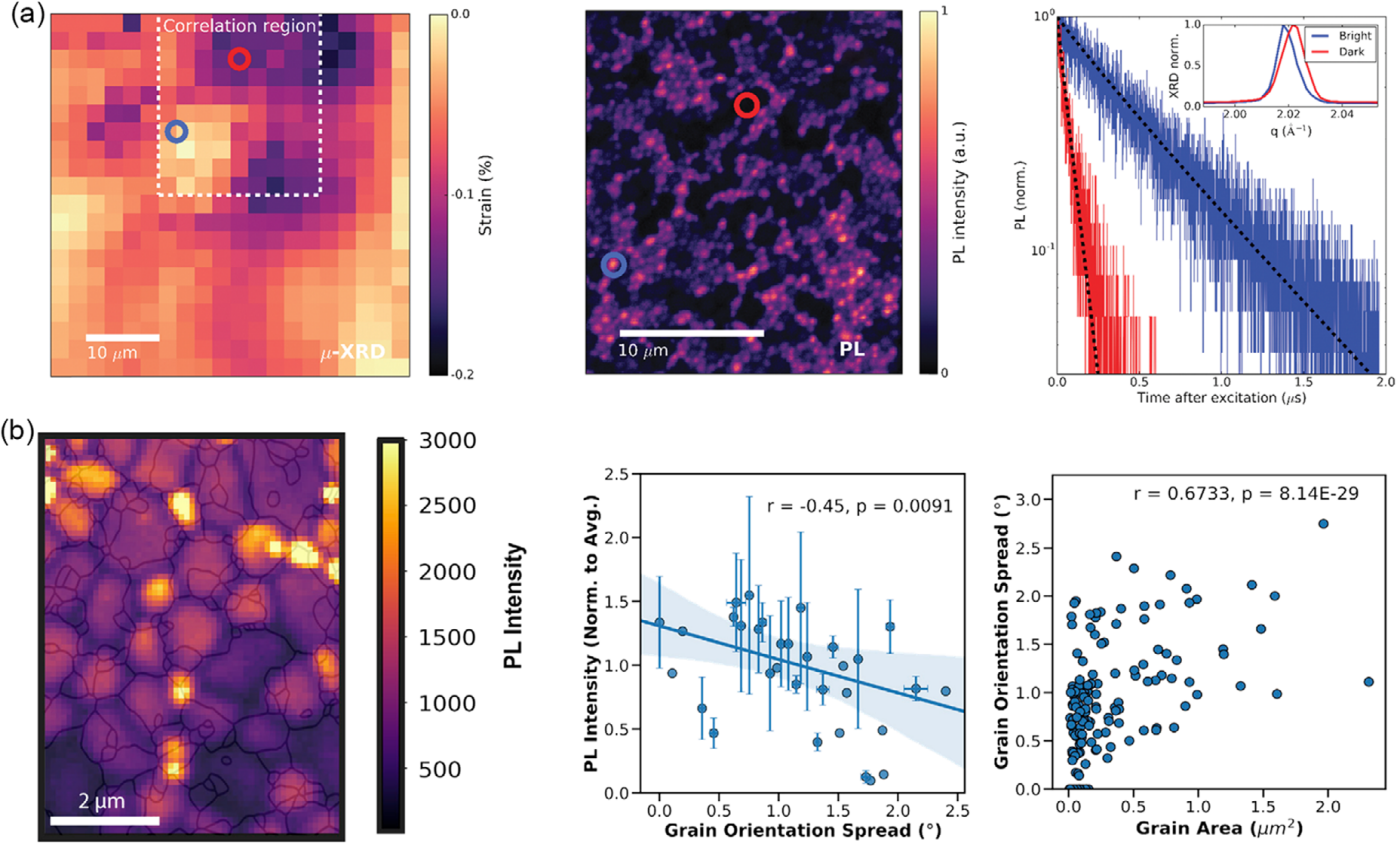
a) Exploring the local structure of MAPbI_3_ films by time‐resolved luminescence properties. Reproduced with permission.^[^ ^11^ ^]^ Copyright 2019, Royal Society of Chemistry. b) Correlation between local luminescence, orientation heterogeneity, and grain area in MAPbI_3_. Reproduced with permission.^[^ ^91^ ^]^ Copyright 2019, Elsevier.

Jones et al. reported that lattice strain is a direct cause of higher nonradiative recombination and defect concentrations, which act as a catalyst for the development of defects during film crystallization and crystal growth.^[^ ^11^ ^]^ They also demonstrated that the dark region relates to the compressive‐strained [220] lattice planes with inferior carrier lifetime dynamics and weak emission intensity, whereas the bright region corresponds to comparatively unstrained processes and longer carrier lifetime (**Figure** **6**a). After translating the strain map into a comparable defect density map using a first‐principles atomic model, they found that there was a significant anticorrelation between the charge carrier lifetime and the ratio of halide vacancies in strained crystals. According to these results, the locally diverse strain is mostly to blame for the local PL heterogeneity that has been observed. As a consequence of the local strain heterogeneity, the perovskite crystal formed flaws such as halide vacancies in addition to other point and extended defects. This resulted in a considerable enhancement of nonradiative recombination. In addition, the results of probing nonradiative recombination in semiconductors made of prototypical halide perovskite using multimodal microscopy correlating electron backscatter diffraction (EBSD) with confocal photoluminescence (PL) show that higher local strain causes higher levels of nonradiative recombination in the compositions that were investigated.^[^ ^91^ ^]^ In locations with high strain, there was a large amount of nonradiative recombination, whereas in parts with low strain, there was high PL intensity (low nonradiative recombination) (Figure 6b).

## 4 Regulation of Strain

**Figure 7:**
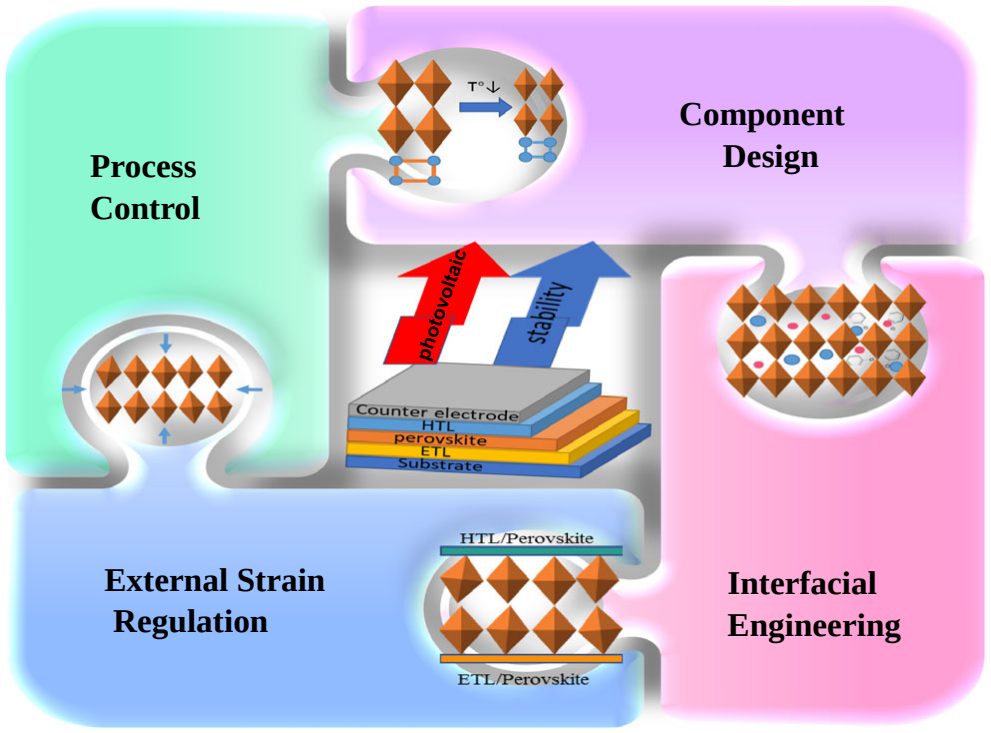
The main strategies to adjust strain.

As was previously mentioned, strain has a significant effect on the stability, optoelectronic characteristics, charge carrier transport, and nonradiative recombination of perovskite films. This can have both positive and negative effects on the performance and stability of photovoltaic solar cells. As a result, a sensible strain‐regulation method is required to improve perovskite film stability. The heart of strain regulation is to reduce defects and increase *E*_a_ of ion migration activation energy, achieving the stable and superior photovoltaic performance ultimately. This section will explain the primary strain adjustment techniques, including process control, component design, interface engineering, and external strain regulation, which are encapsulated in **Figure** **7**.

### 4.1 Process Control

**Figure 8:**
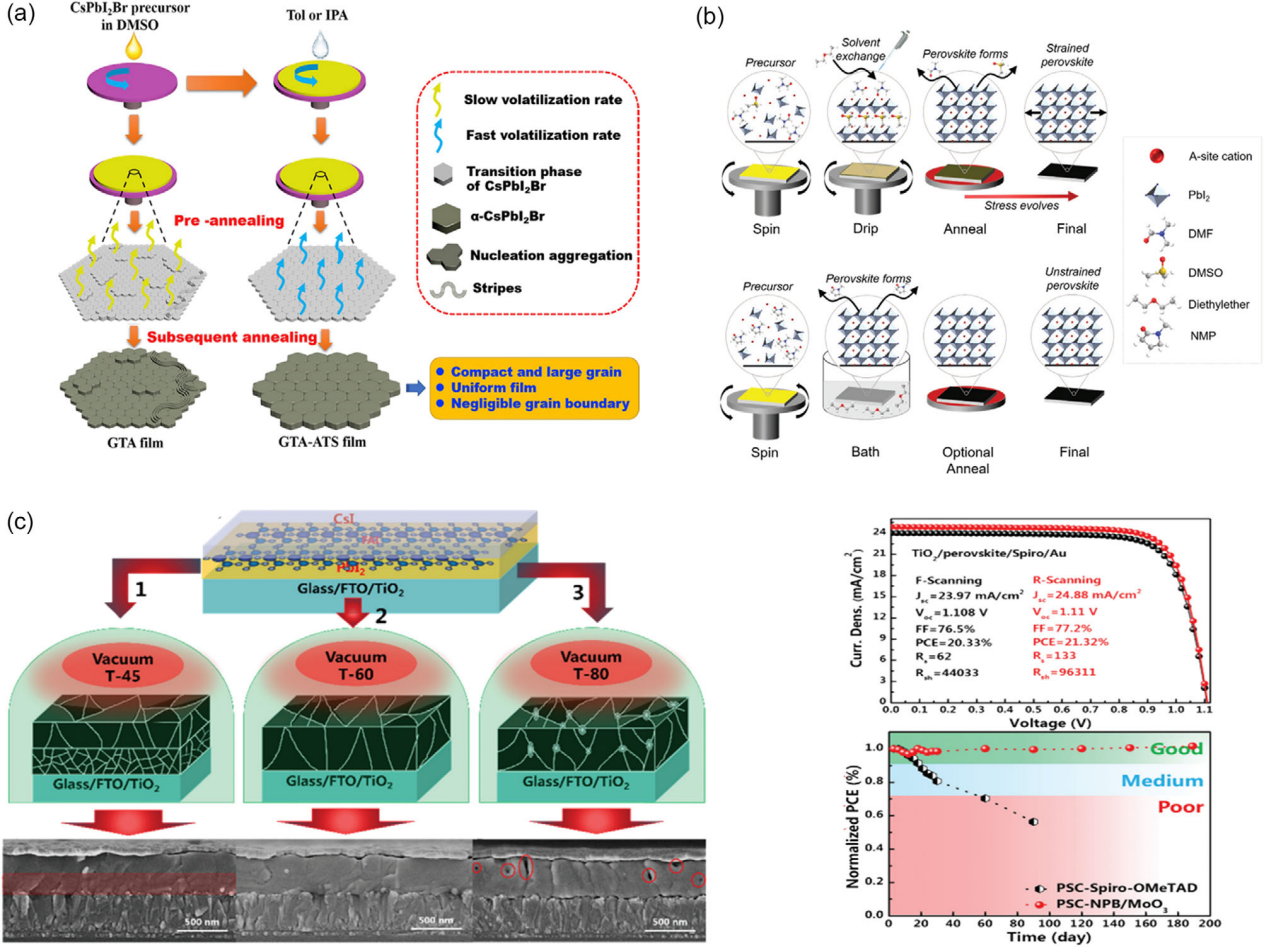
a) The crystallization process of CsPbI_2_Br perovskite via gradient thermal annealing (GTA) or GTA combined with antisolvent (GTA–ATS) processing. Reproduced with permission.^[^ ^94^ ^]^ Copyright 2019, Elsevier. b) The formation stages of perovskite films of antisolvent strategy and the bath conversion method. Reproduced with permission.^[^ ^96^ ^]^ Copyright 2018, Wiley‐VCH. c) Schematic diagram of FA‐based prelayers by vacuum deposition with annealing temperatures of 45, 60, and 80 °C and the corresponding cross‐sectional scanning electron microscopy images. The *J*–*V* curve and the corresponding environmental stability of the unencapsulated PSCs under 35% humidity in the dark. Reproduced with permission.^[^ ^97^ ^]^ Copyright 2021, Royal Society of Chemistry.

Unintentional flaws in perovskite materials are inexorably created during the nucleation and crystallization stages of the crystal development process due to their easy solution processability. The precursor solvent's supersaturation condition closely relates to the supersaturated state that triggers nucleation, and solvent evaporation drives the subsequent growth of the crystal grain. So, it is important to start by taking into account solvent engineering or solvent selection as a fabrication process. During the spin‐coating process, the antisolvent treatment efficiently causes quick supersaturation, which helps to speed up the nucleation and provides a high crystallization layer with a smooth surface. In most cases, annealing is necessary to evaporate any remaining antisolvent, such as chlorobenzene (CB), which leads to tensile strain when the material cools to ambient temperature. Some green antisolvents with low boiling points, such as ethyl acetate (EA), isopropanol (IPA), dichloromethane (DCM), toluene (TOL), and diethyl ether (DEE), have been used to inactivate the crystal defects and optimize the morphology of inorganic perovskite films in order to reduce the strain brought on by the high annealing temperature or residual solvent.^[^ ^92^ ^]^ A high‐quality inorganic CsPbI_2_Br perovskite absorber was created by Chen et al.^[^ ^93^ ^]^ using the EA antisolvent treatment that sped up the film production process. Due to its adequate volatility and viscosity, Chen et al.^[^ ^94^ ^]^ found that IPA could remove the leftover DMSO and lessen the edge solvent impact (**Figure** **8**a).

Besides, reducing the annealing temperature of perovskite film or choosing a substrate with a thermal expansion coefficient comparable to that of the perovskite film is an effective way to relax the tensile strain.^[^ ^95^ ^]^ According to Rolston and colleagues’ findings (Figure 8b), a complete perovskite film with low stress can be formed without annealing by a bath conversion method, submerging the spin‐coated perovskite film in diethyl ether solvent.^[^ ^96^ ^]^ Feng et al.^[^ ^97^ ^]^ revealed an efficient low‐temperature annealing deposition technique in vacuum that works with large‐area, high‐throughput processing. Superior FA‐based perovskite films could be produced in the high vacuum at annealing temperatures of 60 °C; perovskite films have large grain size, good crystallinity, strong PL intensity, high photoelectric conversion efficiency, and long‐term stability (Figure 8c), indicating that the suppressed trap‐assisted nonradiative recombination and reduced defects mainly result from the strain release. Furthermore, in place of the frequently used fluorine‐doped tin oxide (FTO) or indium tin oxide (ITO) glass substrates, a promising approach to reduce the mismatch between the perovskite and the substrate is to use a substrate with a higher thermal expansion coefficient, such as acrylic resin, polycarbonate, or PET.^[^ ^12^, ^98^ ^]^

### 4.2 Component Design

Intrinsically, the perovskite films are also highly susceptible by lattice deformation due to the polycrystalline nature and soft lattice characteristics, resulting in strain and inducing the material degradation. Thus, structure optimization or component engineering may be used to increase the inherent and environmental stability of perovskite. The composition alloying by octahedron modulation got the most remarkable achievements. A key factor in avoiding the undesirable phase change, suppressing ion migration, and improving the intrinsic stability of perovskite is optimizing the [PbI_6_]^4−^ octahedral structure or minimizing the intragrain strain brought on by octahedral distortion.^[^ ^22^ ^]^ The main strategies include the A‐site cation regulation, B/X site adjustment, and additive in perovskite composition.

#### 4.2.1 A‐Site Regulation

**Figure 9:**
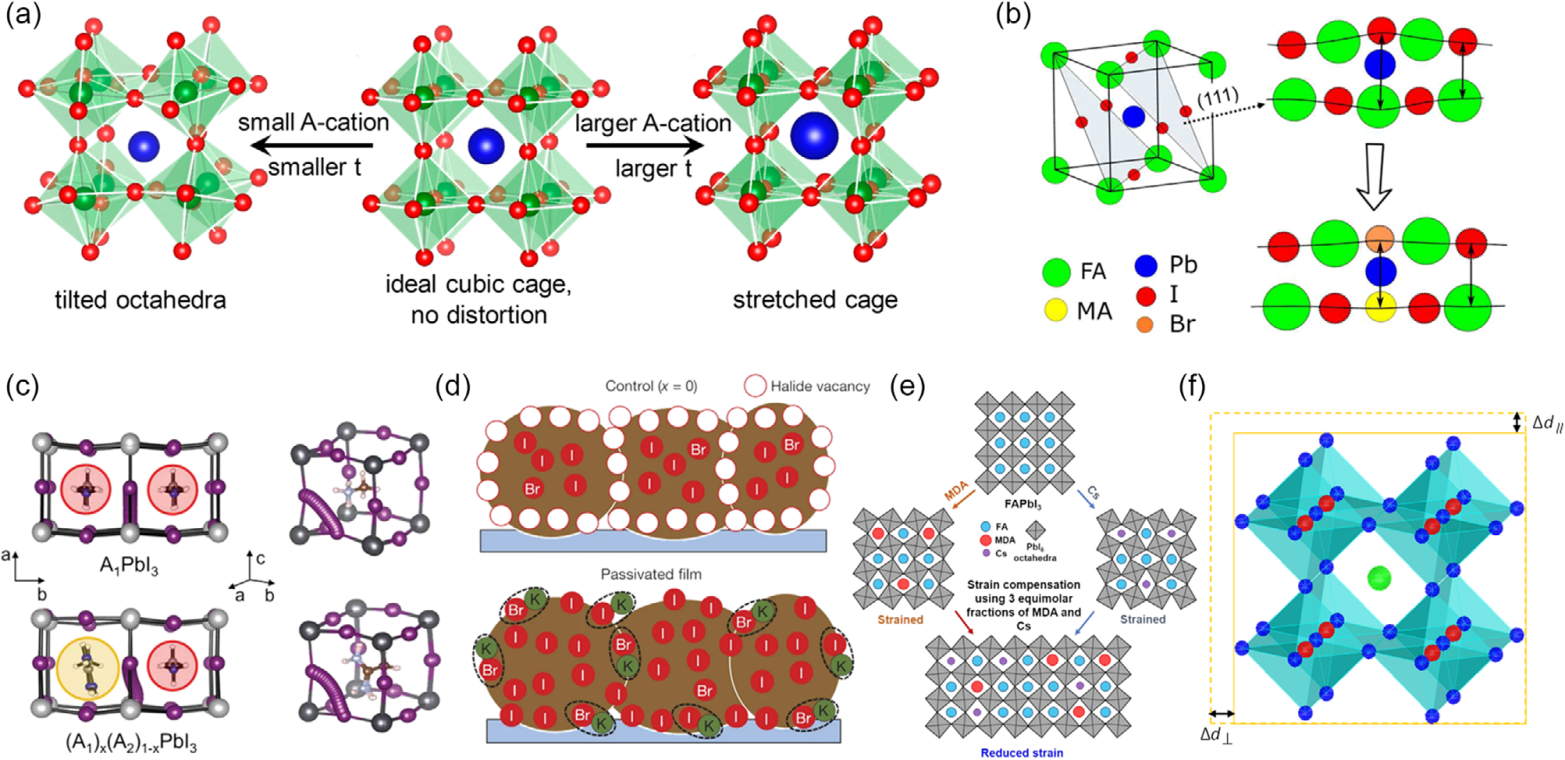
a) Crystal structures of ABX_3_ perovskites with different sizes (smaller, ideal, or larger) of A‐site cation. Reproduced with permission.^[^ ^99^ ^]^ Copyright 2021, American Chemical Society. b) The strain relaxation of FAPbI_3_ after MABr alloying (side view). Reproduced with permission.^[^ ^24^ ^]^ Copyright 2016, American Chemical Society. c) For single‐cation and mixed‐cation compositions, the beginning and final locations of the iodide ion throughout the migration route are defined by the density functional theory model. Reproduction permitted.^[^ ^101^ ^]^ Copyright 2020, Wiley‐VCH. d) The halide‐vacancy management with excess halide, the surplus halide is locked by potassium into benign compounds at the GBs and surfaces. Reproduced with permission.^[^ ^102^ ^]^ Copyright 2018, Springer Nature. e) Schematic illustration of compensating the lattice strain using MDA^2+^ and Cs^+^ cations. Reproduced with permission.^[^ ^6^ ^]^ Copyright 2020, American Association for the Advancement of Science. f) The MA(1Zn:100Pb)I_3−*x*_Cl_*x*_ perovskite realizes the equivalent shrinkage in vertical and horizontal directions, simultaneously. Reproduced with permission.^[^ ^10^ ^]^ Copyright 2018, Elsevier.

As the fillers for inorganic frameworks, the size of A‐site cations determines the distortion degree and tilting level of [PbX_6_]^4−^ octahedral (**Figure** **9**a), significantly influencing the intrinsic properties (such as phase stabilities, photoelectric performance) of perovskites.^[^ ^99^ ^]^ Obviously, the FA^+^ proportions in recent record‐breaking PSCs increased continuously.^[^ ^2^ ^]^ For the pure FAPbI_3_, the Goldschmidt tolerance factor (*t*) value is at the edge of cubic phase due to the big size of FA^+^. As a result, the FAPbI_3_ crystal under anisotropic lattice strain would undergo restructuring, and the environment moisture easily accelerates degradation of the *α*‐black phase (cubic phase) to the yellow phase (*δ* phase/hexagonal phase). Initially, the MA^+^ substitution was widely used to prevent the phase transition of FAPbI_3_, using the MABr or MAI to partially replace the FAI in the precursor solution.^[^ ^24^, ^100^ ^]^ Excitedly, using MABr alloying or FAPbI_3_–MABr is an effective way to balance the lattice strain in (111) plane due to lattice contraction (Figure 9b), thereby preventing the phase transition from *α*‐phase to *δ*‐phase successfully, leading to the remarkably improved stability of *α*‐FAPbI_3_.^[^ ^24^ ^]^ Compared to single‐cation compositions, mixed‐cation compositions are observed to moderate the devices’ hysteresis. This is mostly due to the steric hindrance effect that distorts the ion migration paths owing to the local lattice mismatch (lattice strain) induced by A‐cations of varying sizes in mixed‐cation compositions (Figure 9c).^[^ ^101^ ^]^ The increased *E*_a_ for ion migration hence contributed to the improvement of the thermal and light stabilities of PSCs. Consequently, partial substitution of FA^+^ in FAPbI_3_ with Cs^+^ and/or MA^+^ is usually adopted to stop ions from migrating and defects from forming, relaxing the intrinsic strain.^[^ ^6^ ^]^ In addition, the undersized alkali cation (such as Rb^+^, K^+^, and Na^+^) doping was often used to hinder the ion migration and ease the local strain in perovskite. The excess and undercoordinated halides can be bonded and immobilized by strongly electropositive alkali cations, preventing the unwanted halide ion migration.^[^ ^16^, ^102^ ^]^ At the same time, halogens that coordinate with alkali metal ions also play a positive role to suppress defects. Abdi‐Jalebi et al.^[^ ^102^ ^]^ introduced the potassium iodide (KI) as excess iodide into the perovskite precursor (Cs_0.06_FA_0.79_MA_0.15_Pb(I_0.85_Br_0.15_)_3_) solutions to compensate the halide vacancies (Figure 9d). The halide vacancies would be filled by the excess halides, inhibiting the nonradiative recombination pathways and reducing local strain level, thereby leading to high performance of PSCs. Furthermore, alkali cation doping enhanced the production energy of mobile halide interstitial defects, according to theoretical simulations.^[^ ^16^ ^]^ The role of alkali cations (incorporation into the lattice A site or keeping immiscible phases) in different perovskite compositions may depend on the different miscibility limits. Moreover, it has been shown that the light‐induced strain indicated before may be reduced or even eliminated to improve photoelectric performance using the mixed‐cation approach.^[^ ^50^, ^103^ ^]^

Additionally, adopting surface‐functionalizing oversized A‐cations was demonstrated to be valid for the modulation of surface energy to modulate the perovskite phases.^[^ ^23^ ^]^ Due to the large size characteristics, bulky ammonium cations like isopropylammonium (IPA), phenethylammonium (PEA^+^), or butylammonium (BA^+^) are unable to be integrated into the crystalline lattice bulk, which spontaneously eject to the grain borders and surfaces. So, controlling the surface A site is an efficient way to regulate the connected [BX_6_]^4−^. Thereby forming the steric obstruction effect to hinder the ion migration and promote the increase of Ea, achieving the further improvement of PSCs’ thermal and photo‐stabilities.^[^ ^104^, ^105^ ^]^ Moreover, using the divalent cation such as methylenediammonium (MDA^2+^) to form a greater number of hydrogen bonds with the [BX_6_]^4−^ lattice is beneficial for stabilizing the cubic phase.^[^ ^106^ ^]^ Nevertheless, the oversized A cation incorporation inadvertently leads to the tensile lattice strain. In view of this, the smaller Cs^+^ cation might be introduced to lessen the remaining stress (Figure 9e).^[^ ^6^ ^]^

#### 4.2.2 B/X‐Sites Regulation

It is vital to note that lead halide perovskite stability is improved by A‐site alloying, but the lattice strain management on the B/X sites must also be taken into consideration. The incorporation of potential dopants in the B/X sites of the CsMAFA‐based perovskites has thus been researched. The addition of Cl^−^ and Cd^2+^ among numerous possibilities demonstrated a notable improvement in stability under varied circumstances.^[^ ^107^ ^]^ According to Shai et al.,^[^ ^10^ ^]^ a moderate zinc (Zn) substitution is advantageous for obtaining the ordered and stable MA(Zn:Pb)I_3−*x*_Cl_*x*_ crystal, releasing lattice strain with a suitable lattice constriction due to the equally balanced horizontal and vertical shrinkage of the BX_6_ octahedron. (Figure 9f). Additionally, effective halide component management helps to improve environmental and intrinsic stability, modify the electronic structure, and get rid of lattice strain.^[^ ^108^ ^]^ It has been shown that adding tiny Cl anions to perovskite lattices prevents the material from breaking down and increases its stability, preventing the light‐induced phase segregation of Cl‐alloyed perovskite and the corresponding PSCs.^[^ ^109^ ^]^ During the annealing process, the reconstruction and mass transport of the perovskite film can be enhanced by chloride incorporating due to its easy escape, eradicating the topical islands and improving the quality of the perovskite film. It significantly reduces the nonradiative recombination and greatly improves the carrier transport across heterojunction interfaces, thereby leading to high PCE and long‐term stability of PSCs.^[^ ^110^ ^]^ Moreover, it has been demonstrated that the suppression of halide segregation with Cl alloying promotes the MHPs to keep their identity in delivering charge carrier separation, which is mainly due to the increasing *E*_a_ for halide ion migration.^[^ ^111^ ^]^ By altering the octahedral structure and reducing the lattice parameter, the partial replacement of iodine with bromine is advantageous for increasing the solubility of chlorine in perovskite crystal and effectively suppressing light‐induced phase segregation.^[^ ^19^ ^]^ Except for halide anions, pseudohalide anions (such as SCN^−^, Ac^−^ and BF_4_^−^, etc.)^[^ ^112^ ^]^ with halogen‐like qualities may partially integrate into the X‐site to change the [PbX_6_]^4^ octahedron and control the natural characteristics of perovskites, resulting in the relaxation of lattice strain. For instance, MAPbI_3−*x*_(SCN)_*x*_ PSCs were made using Pb(SCN)_2_ precursor. SCN^−^ was successfully used to passivate the flaws and increase the inherent stability of perovskite films by being absorbed into the crystal lattice.^[^ ^113^ ^]^ The tetrafluoroborate (BF_4_^−^) anions were proven to enter (FAPbI_3_)_0.83_(MAPbBr_3_)_0.17_ perovskite crystal frame; the performance of PSCs and other optoelectronic devices may be improved by films that have longer PL lifetimes, reduced trap densities, and lattice relaxation.^[^ ^114^ ^]^

#### 4.2.3 Additive Engineering

**Figure 10:**
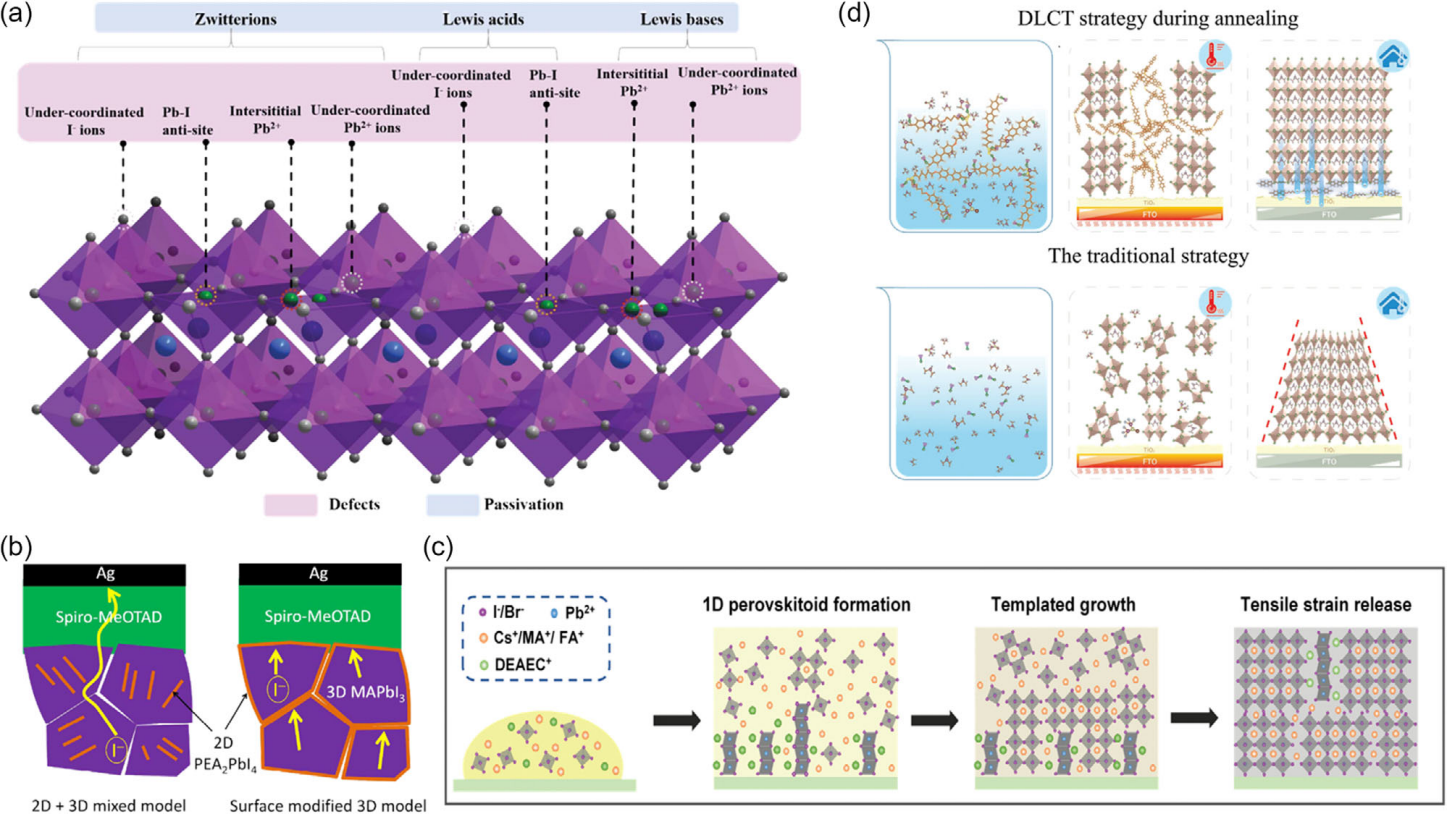
a) The strategies of defect passivation to release strain by different Lewis additives (Lewis acids, Lewis bases, and zwitterions). Reproduced with permission.^[^ ^117^ ^]^ Copyright 2020, Royal Society of Chemistry. b) Schematic illustrating of the suppressed ion migration by GB 2D (PEA)_2_PbI_4_. Reproduced with permission.^[^ ^123^ ^]^ Copyright 2017, American Chemical Society. c) Schematic diagram of the tensile strain relaxation during the growth process of 1D@3D perovskite films. Reproduced with permission.^[^ ^4^ ^]^ Copyright 2021, Wiley‐VCH. d) Diagram of the defects passivation and tensile stress relaxation by DLCT strategy. Reproduced with permission.^[^ ^125^ ^]^ Copyright 2022, Wiley‐VCH.

Engineering with additives has been frequently employed to passivate undesirable defects on the surfaces or GBs of perovskite films. Recently, some organic molecules or polymers have been used to regulate the strain of perovskite films. For instance, it has been confirmed by Li et al. that appropriate β‐poly(1,1‐difluoroethylene) in perovskite solution is beneficial to reduce the thermal strain through crystallization control and energy arrangement.^[^ ^115^ ^]^ Additionally, additives like Lewis acid, Lewis base, and zwitterions to coordinate the undesirable defects have also been explored. The dative Lewis adducts significantly contribute to the relaxation of the interface strain.^[^ ^89^, ^116^ ^]^ As depicted in **Figure** **10**a, different Lewis additives were used to form coordination interaction with different charged defects to release strain.^[^ ^117^ ^]^ Lewis base additives (such as N‐donor, O‐donor, S‐donor, and P‐donor) were employed to passivate the positively charged defects (such as undercoordinated Pb^2+^ ions and interstitial Pb^2+^).^[^ ^118^, ^119^ ^]^ Lewis acids, such as fluorine‐containing aromatic molecules (iodopentfluorobenzene (IPFB), tris(pentafluorophenyl)phosphine (TPFP)), fullerene, and its derivatives (such as [6,6]‐phenyl‐C61‐butyric acid methylester, PCBM) were employed to inactivate the negatively charged defects (such as Pb–I antisites and under‐coordinated halide ions).^[^ ^120^, ^121^ ^]^ The zwitterions, such as 3‐(1‐pyridinio)‐1‐propanesulfonate, have a good bilateral action that may concurrently cure defects that are negatively and positively charged.^[^ ^119^, ^122^ ^]^

Meanwhile, using low‐dimensional (LD) perovskite to create an LD/3D heterostructure is a tried‐and‐true way to further boost stability by releasing surface strain. In order to reinforce and safeguard the 3D perovskite lattice, for instance, using the larger organic A cations to create 2D/3D structures is a successful technique to cause steric hindrance, which retards the heat breakdown of hydrogen bonding and prevents ion movement (Figure 10b).^[^ ^123^, ^124^ ^]^ Additionally, Kong et al. formed highly crystalline 1D@3D perovskite films with improved stability and reduced residual tensile strain alleviative effects using 2‐diethylaminoethylchloride hydrochloride (DEAECCl) to manufacture 1D perovskitoid (DEAECPbI_3_) with excellent charge transfer capability (Figure 10c).^[^ ^4^ ^]^

Furthermore, Du et al.^[^ ^125^ ^]^ developed a dynamic liquid‐crystal transition (DLCT) strategy by designing a thermotropic liquid crystal molecule (CBO6SS6OCB), leading to reduced defects and released tensile stress during the fast bulk crystallization in one step (Figure 10d). First off, the intermediate adduct caused by the interaction between LC molecule and perovskite colloid retarded the crystallization of perovskite and resulted in big grains. Second, during the annealing procedure, the concentrated LC solid was stimulated to flow to the electron transport layer, and the concentrated molecules interacted with the carrier transport layer and acted as a buffer to overcome the thermal mismatch and relieve the remaining strain between the ETL and perovskite layer. Similarly, Zhang et al. introduced the crosslinking‐enabled strain‐regulating crystallization (CSRC) method based on trimethylolpropane triacrylate (TMTA) to adjust the lattice distortion on the top of perovskite film. This in situ CSRC method acts as a synergy effect on defects passivation, strain regulation, and humidity exclusion, thereby leading to PSCs with high efficiency and long‐term photostability.^[^ ^126^ ^]^ In addition, they also apply 1‐butyl‐3‐methylimidazolium‐based ionic liquids to react with excess PbI_2_, achieving the Pb defects passivation and tensile strain release, resulting in the improvement of efficiency and long‐term stability of PSCs.^[^ ^127^ ^]^

### 4.3 Interfaces Engineering

Interfacial residual stresses would emerge from the mismatched thermal expansion coefficients between perovskite films and carrier transport layers, as mentioned in Section 2.2.1. Even though a substrate with a higher thermal expansion coefficient or low‐temperature perovskite layer preparation are advantages to reduce the mismatch between the substrate and perovskite, it is typically extremely difficult to prepare high‐quality perovskite films based on the aforementioned strategies.^[^ ^13^ ^]^ Therefore, it is necessary to release the residual strain through developing appropriate interface materials to modify ETL/perovskite and perovskite/HTL interfaces. Recently, to regulate interfacial residual strain, interface layers with different thermal expansion coefficients have been employed.^[^ ^12^ ^]^

#### 4.3.1 ETL/Perovskite Interface

**Figure 11:**
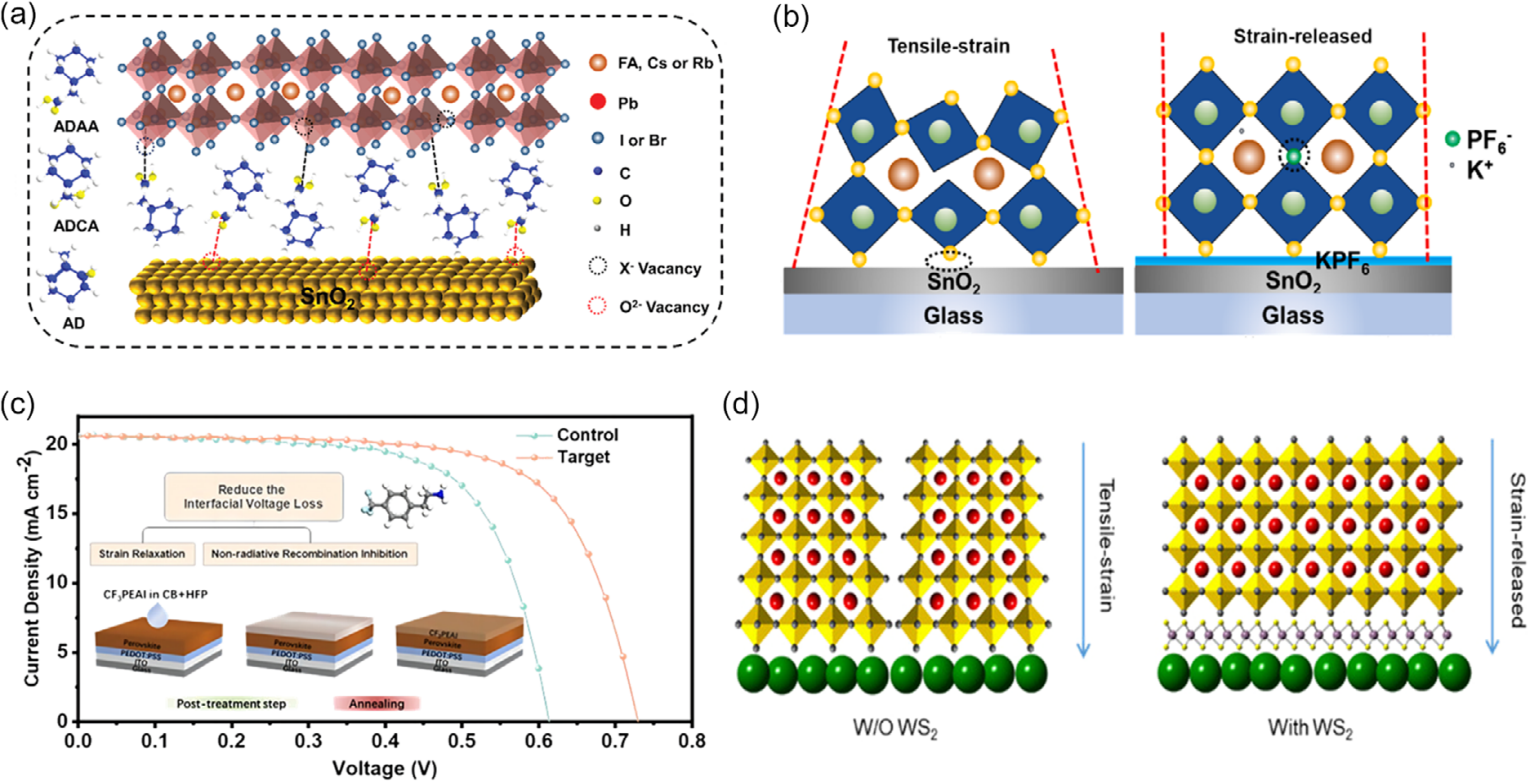
a) Molecular structures of AD, ADCA, and ADAA modifiers, and the diagram of interfacial defect passivation by them. Reproduced with permission.^[^ ^18^ ^]^ Copyright 2022, Wiley‐VCH. b) The mechanism diagram of residual strain in perovskite films prepared on the undecorated SnO_2_ and KPF_6_‐modified SnO_2_. Reproduced with permission.^[^ ^17^ ^]^ Copyright 2021, Elsevier. c) The effect diagram on photovoltaic of PSCs by CF_3_PEAI. Reproduced with permission.^[^ ^128^ ^]^ Copyright 2022, Elsevier. d) The effect diagram of strain relaxation in CsPbBr_3_ grains by WS_2_ interlayer. Reproduced with permission.^[^ ^129^ ^]^ Copyright 2020, Wiley‐VCH.

To modify the SnO_2_/perovskite interface, Zhou et al.^[^ ^18^ ^]^ used a variety of adamantane derivative molecules functionalized with C═O, including 2‐adamantanone (AD), 1‐adamantane carboxylic acid (ADCA), and 1‐adamantaneacetic acid (ADAA). All of them contributed to the reduction of interfacial defects, the release of interfacial strain, and the enhancement of device performance (**Figure** **11**a). By adjusting the distance between the bulky adamantane ring and C═O, it is possible to control the steric barrier of chemical interaction between C═O and SnO_2_ as well as perovskite. The interfacial strain release mostly benefits from the interface defect passivation, and the defect passivation impact is inversely proportional to the steric hindrance. As a result, the ADAA‐based PSCs by the two‐step‐coating procedure obtained a champion efficiency of 23.18%. The KPF_6_ molecule was used by Bi et al.^[^ ^17^ ^]^ to alter the SnO_2_/perovskite interface. It was shown that PF_6_^−^ was present at the interface to passivate interface flaws, whereas the majority of K^+^ ions diffused into the perovskite layer to passivate defects that were negatively charged. Due to the hydrogen interaction between PF_6_^−^ and perovskites and the coordination bond between PF_6_^−^ and SnO_2_, the interfacial contact was enhanced. The interfacial defect passivation and interfacial strain release brought about by KPF_6_ alteration increased PCE from 19.66% to 21.39% (Figure 11b).

Furthermore, an extra 2D layer is often introduced at the ETL/perovskite interface to increase surface hydrophobicity, prevent surface recombination, and minimize defect states, thereby to generate the stable PSCs with high efficiency. Chen^[^ ^128^ ^]^ et al. introduced phenylethylamine hydroiodide derivative 3‐(trifluoromethyl)phenylethylamine hydroiodide (CF_3_PEAI) to form 2D layer (CF_3_PEA)_2_SnI_4_ to suppress the charge carrier nonradiative recombination at the perovskite/PC_61_BM interface, and the GBs of perovskite films were reduced by the introduction of the large organic cation. This surface reconstruction is beneficial to relax the surface tensile strain and reduce the trap density (Figure 11c). As shown in the Figure 11d, the perovskite tensile strain was effectively released and the lattice became more compact due to the 2D WS_2_ deposition on SnO_2_–TiO_*x*_Cl_4−2*x*_ to form the lubricant layer between ETL and CsPbBr_3_ perovskite, resulting from atomically smooth dangling bond‐free surface of WS_2_ nanoflake.^[^ ^129^ ^]^

#### 4.3.2 Perovskite/HTL Interface

**Figure 12:**
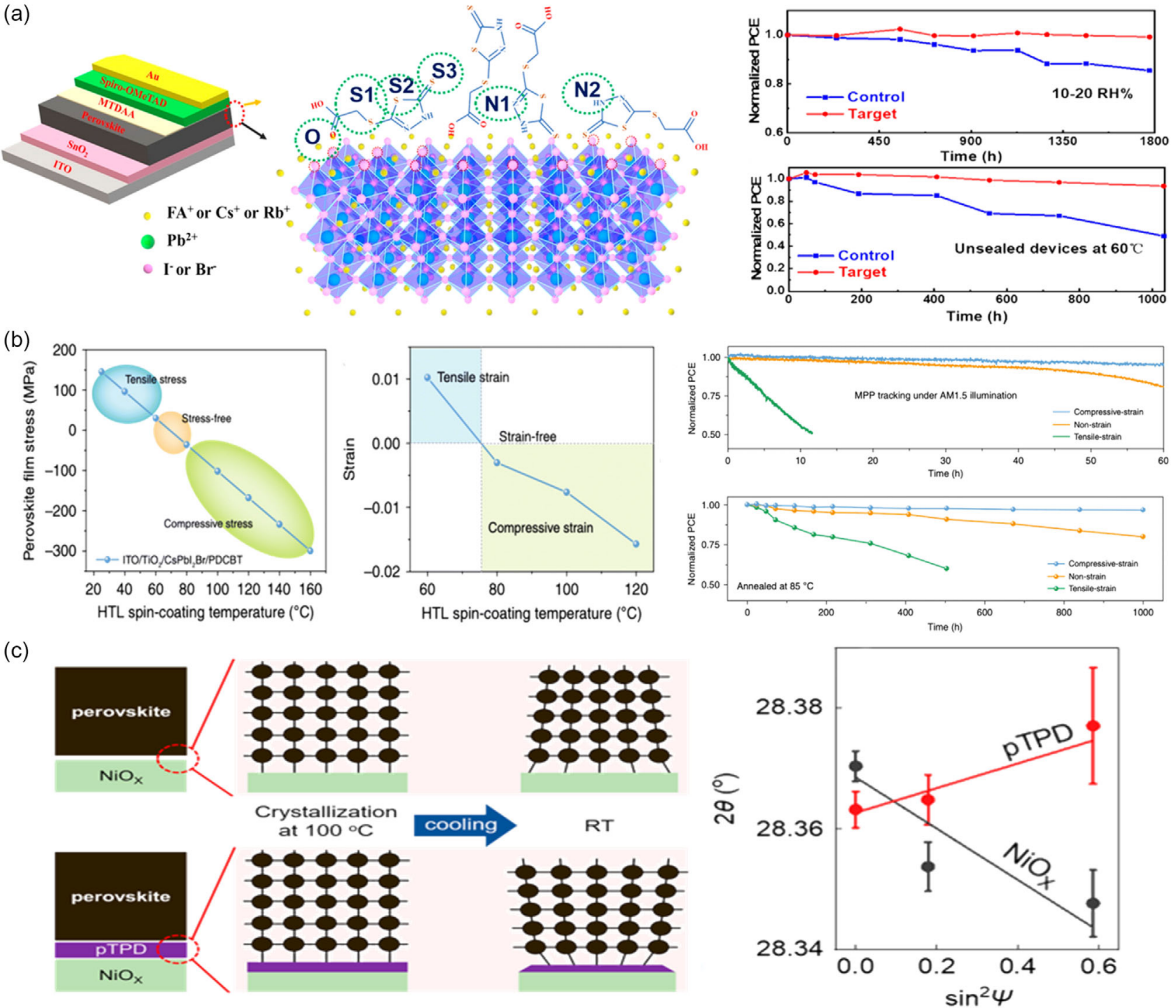
a) The defect passivation of MTDAA on PSCs and the superior environmental and thermal stability. Reproduced with permission.^[^ ^134^ ^]^ Copyright 2021, American Chemical Society. b) Calculated and measured stress variation of CsPbI_2_Br vary from the HTL (PDCBT) spin‐coating temperature, and the light and thermal stability of the devices with various strains under MPP tracking. Reproduced with permission.^[^ ^12^ ^]^ Copyright 2020, Springer Nature. c) Schematic diagram of residual strain in perovskite films which undergo the annealing process on NiO_*X*_ and pTPD, and shift of the perovskite crystals peak position corresponding to the (002) plane as a function of sin2ψ on NiO_*X*_ and pTPD. Reproduced with permission.^[^ ^136^ ^]^ Copyright 2022, American Chemical Society.

The PCE and stability of PSCs are significantly increased by passivation for the electronic imperfections present in the bulk of the perovskite and at the perovskite‐hole conductor contact.^[^ ^130^ ^]^ Some molecular modulators such as 1‐adamantylamine (ADA),^[^ ^131^ ^]^ cyclohexylmethylammonium iodide (CMAI), ammonium salts,^[^ ^132^ ^]^ and modified NiO_*x*_^[^ ^133^ ^]^ were demonstrated effectively to passivate the uncoordinated Pb^2+^ defects through coordination interactions.

(5‐Mercapto‐1,3,4‐thiadiazol‐2‐ylthio)acetic acid (MTDAA) was used by Liu et al.^[^ ^134^ ^]^ to modify the surface and GBs of MA‐free perovskite films. The uncoordinated Pb^2+^ defects at GBs or the surface of perovskite films were strongly interacted with by the multiple active sites in MTDAA molecules, resulting in a reduction in defect density, an extension of the carrier lifetime, and a thorough release of interfacial residual stress. Excellent long‐term stability was shown by the MTDAA‐modified unencapsulated device. (**Figure** **12**a). Similarly, Shi et al. used the symmetric 1,1′‐(methylenedi‐4,1‐phenylene)bismaleimide (BMI) molecule to toughen the interface through chemical bonding with the uncoordinated Pb^2+^ or FA^+^ simultaneously, thus significantly enhancing the intrinsic stability of perovskite films.^[^ ^135^ ^]^

Furthermore, by adding an external compressive strain from the HTL with a high thermal expansion coefficient, it is possible to counteract the residual tensile strain brought on by the mismatched lattice in PSCs. In order to lessen the tensile strain in perovskite films, Xue et al.^[^ ^12^ ^]^ explored a strain compensation technique based on the use of ([5,5‐bis(2‐butyloctyl)‐(2,2bithiophene)‐4,4'‐dicarboxylat‐*alt*‐5,5'‐2,2'‐ bithiophene](PDCBT)) with thermal expansion coefficient of 31.5 × 10^−5^ K^−1^ (more than an order of magnitude higher than that of CsPbI_2_Br). A strong HTL/perovskite interface was created by the strong interactions between the carbonyl groups of PDCBT and the perovskite surface. This interface successfully transferred compressive strain from the HTL to the perovskite active layer, complementing the temperature tensile strain in the perovskite layer. Additionally, it was discovered that perovskite thin‐film devices both with and without compressive strain exhibit remarkable durability under continuous sunshine and high temperature (80 °C). This study demonstrates how changing the HTL may transform residual tensile strain perovskite films into compressive strain or strain‐free perovskite films, which are more stable. After recording the highest power point for 60 and 1000 h at 85 °C, the resultant photovoltaic devices still had 95% (16.4%) of their original PCE (Figure 12b). Moreover, adding a higher *α* interlayer may both reduce the tensile strain on the perovskite and conversely produce a compressive strain at the interface by placing it between the perovskite and HTL layer. By leveraging a thermal expansion differential between the perovskite layer and substrate, Ju et al.^[^ ^136^ ^]^ modified the approach to apply compressive strain into PSCs. They used a polymer called poly(4‐butylphenyldiphenylamine) that has a greater thermal expansion coefficient than perovskite as a substrate for perovskite crystal growth at 100 °C and then cooled to room temperature. With the the greater contraction of the polymer, compressive strain was successfully introduced into perovskite film in the annealing and cooling process (Figure 12c).

### 4.4 External Strain Regulation

It is possible to increase the optoelectronic property and stability of PSCs by controlling the strain externally, including the pressure assisted by changing the type or degree of pressure, as well as light management schemes through intensity and density changes.^[^ ^14^, ^137^, ^138^, ^139^, ^140^, ^141^, ^142^ ^]^ Following is a methodical discussion mainly from pressure‐assisted and light soaking.

**Figure 13:**
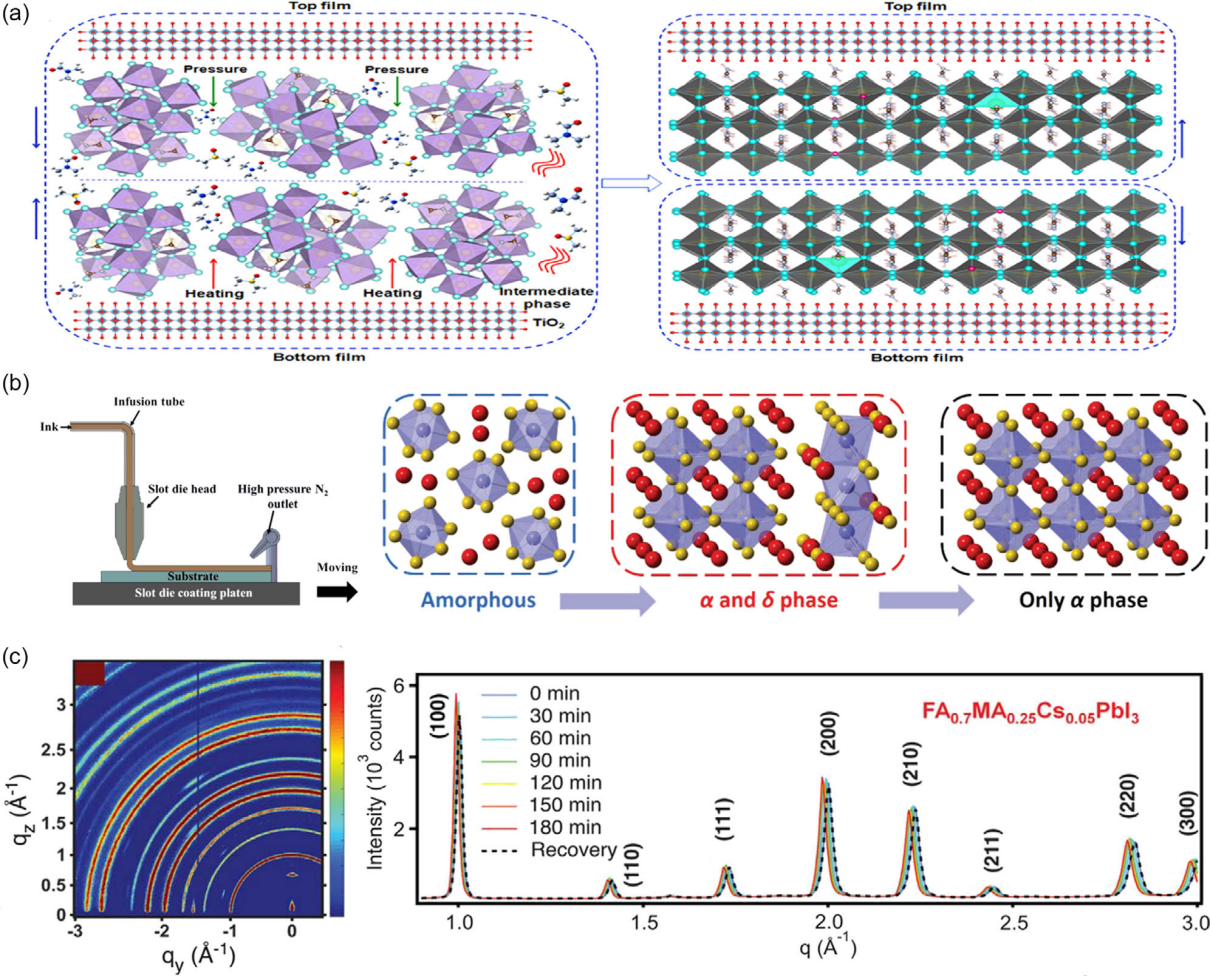
a) Schematic of modulation of strain in perovskite films via PASP method. Reproduced with permission.^[^ ^137^ ^]^ Copyright 2020, Elsevier. b) Schematic illustration of the route of slot‐die coating with HPNE, and the evolution of the corrosponding perovskite film by this fabrication. Reproduced with permission.^[^ ^14^ ^]^ Copyright 2020, Wiley‐VCH. c) The GIWAXS picture and the X‐ray diffraction peaks of the cubic FA_0.7_MA_0.25_Cs_0.05_PbI_3_ films under different illumination time. Reproduced with permission.^[^ ^50^ ^]^ Copyright 2018, Springer Nature.

Strain is a key enabler to design a stable optoelectronic device, the matching degree between the imposed axial stress and elasticity modulus in the corresponding crystal direction determines the lattice and bandgap of the perovskite, and an appropriate regulation is urgently needed.^[^ ^143^ ^]^ As expected, Steele et al. developed the substrate clamping and biaxial strain to form the stable black‐phase CsPbI_3_ thin film at room temperature.^[^ ^144^ ^]^ In order to study the relationship between the pressure and their contact interface, Oyelade et al.^[^ ^140^ ^]^ applied a range of pressures (0–10 MPa) on the fabricated PSCs. They confirmed that the pressures of 0–7 MPa were beneficial for the improvement of PSCs’ photoconversion efficiencies, and the pressure beyond ≈7 MPa was detrimental to device performance. Additionally, pressure‐assisted solution processing (PASP), which allows for the regulation of strain, enables the control of perovskite crystal nucleation and growth.^[^ ^137^ ^]^ With micrometer‐sized grains, carrier lifetimes in the microsecond range, decreased intrinsic ion migration, and improved extrinsic light and moisture stability, high‐quality perovskite layers may be produced using this method. **Figure** **13**a depicts the PASP production process for perovskite films. After aging for 60 days and long‐term (200 h) continuous 1‐sun illumination in ambient environment without encapsulation, the related solar cell achieved a champion PCE of 20.74% with minimal *J*–*V* hysteresis, keeping over 90% of the starting PCE. Moreover, gas quenching is a potential method for up‐scale production of perovskite films in practical applications, which is suggested to replace the antisolvent quenching in order to prevent the detrimental effects from the antisolvents.^[^ ^145^ ^]^ Due to adjustable supersaturation, the crystallization process can be carried out with extreme precision, resulting in great repeatability and resilience in the production of high‐quality perovskite films. According to Hou et al.,^[^ ^139^ ^]^ a low‐gas‐pressure accessible gas‐quenching approach to create MA‐ and Br‐free PSCs with no passivation treatment has a PCE of 21.3%. Furthermore, the method of high‐pressure nitrogen extraction (HPNE) was used to deposit superior perovskite absorber layers at room temperature,^[^ ^14^ ^]^ resulting in a printing process that has a broad processing window and is essentially independent of the coating speed, liquid flow rate, and N_2_ flow pressure. The films that underwent HPNE processing had an incredibly compact and homogenous morphology made up of big grains that had an average size of 580 nm. There are two reasons why this improvement may be made. The high‐speed nitrogen flow, for starters, encourages the speedy evaporation of solvents from the wet coating, thus bringing the system to an oversaturated condition for homogenous crystallization. Second, the rapid gas expansion from high pressure to atmospheric pressure causes the compressed nitrogen to escape from the outlet into the surrounding air, causing a large temperature decrease (the coated surface from 25.0 to 12.9 °C), which causes oversaturation for uniform crystallization. The schematic depiction of the preparation process and development of perovskite film using slot‐die coating and HPNE is shown in Figure 13b.

On the other hand, the light‐induced structure change is crucial to control the optoelectronic capabilities and stability of perovskite devices. To produce the uniform lattice expansion perovskite films with big crystals, Tsai et al. employed continuous light soaking with a conventional 1‐sun (100 mW cm^−2^). Any phase segregation or degradation under continuous lighting time from 0 to 180 min wasn't found. According to Figure 13c's plot, the diffraction peaks evenly moved in the direction of smaller scattering vector **q** values. Resulting from the light‐induced uniform lattice expansion, the average structural correlation length is increased, leading to a reduction in the energetic barrier that is present near the perovskite contact interfaces and weakening of the nonradiative recombination process. This releases the local strain and improves the open‐circuit voltage and fill factor, achieving the enhancement of PSCs’ stability and PCE.^[^ ^50^ ^]^ Furthermore, Rolston et al.^[^ ^103^ ^]^ confirmed that the lattice expansion under illumination is indeed consistent with heat‐induced thermal expansion, actually by precisely regulating the temperature during the illumination process and strengthening the understanding for external strain regulation of light soaking.

## 5 Summary and Perspective

### 5.1 Summary

The scientific community is paying more attention to strain in metal halide perovskite and PSCs. A thorough knowledge of the causes and effects of strain is crucial and necessary to produce the stable and effective PSCs. In this article, we carefully describe the causes, the strain's impact on the physical characteristics and photovoltaic performance, as well as the solutions for reducing it to improve the photoelectricity and stability of perovskite films and solar cells.

Strain in perovskite is normally caused by: 1) internal strain induced by the symmetry disruption due to the octahedra tilting or complicated crystallization (heterogeneous phase) of perovskite crystal and 2) external strain generated by the lattice periodicity distortion resulting from the lattice/*α* mismatch or external conditions such as illumination, electrical bias, temperature and external pressure, etc.

The effects of strain on the physical characteristics and photovoltaic performance of perovskites and solar cells have also been summarized, with particular attention paid to how they affect physical stability processes like ion migration and phase segregation as well as carrier transport and nonradiative recombination. 1) In perovskites, strain has a synergistic influence on the phase segregation caused at various length scales, from the atomic to the macrolevel, via affecting ion movement and resulting in undesirable phase segregation. 2) Strain affects the bandgap and charge carrier mobility by altering the crystal structure. More stable films would arise from the compressive strain (alleviating tensile strain), but it is difficult to get compressive strain without associated tensile strain due to Poisson's effect. 3) Generally, under tensile strain, local strain heterogeneity is common in perovskite films. On the contrary, compressive strain is advantageous to increase the charge carrier lifetime and improve the inherent stability of perovskites.

Based on earlier understanding, the following list of strain regulation techniques has been put together to enhance the photovoltaic efficiency and long‐term stability of PSCs. 1) Processing engineering via saturation control, lower‐temperature preparation, and lattice or thermal expansion coeﬃcient matching between the adjacent semiconductors are the main strategies to reduce the detrimental lattice strain during crystal formation. 2) Composition engineering on the perovskite layer through A‐site or B/X‐sites alloying and additive in perovskite engineering is an optimization strategy. 3) Interfacial engineering on ETL/perovskite or perovskite/HTL interface via the intervention from extra interlayers is another technique. 4) Engineering of external strain from environmental stressors including light, air pressure, and temperature is another technique.

### 5.2 Perspective

Halide PSCs have PCEs that are close to 26%. The difficulties with long‐term stability, however, prevent their commercialization. According to recent research, the stability and efficiency of devices are largely influenced by the strain present in halide perovskites. Regulating the compressive strain is an effective method to improve the optoelectronic capabilities and stability of perovskite. However, it is difficult to get the compressive strain without associated tensile strain due to Poisson's effect, and the local strain heterogeneity is prevalent in the perovskite films. Therefore, residual strain release or achieving strain‐free perovskite films is the ultimate aim of strain regulation. Based on the existing cognition, we propose the following pathways. 1) Developing novel or modified methods for producing high‐quality perovskite films, with a particular emphasis on lattice or *α‐*matching via preparation at low temperatures or high‐*α* *‐*substrate investigation, is the main strategy to reduce the detrimental lattice strain during crystal formation. 2) Based on the defect passivation and intrinsic stability improvement by alloying or additive in perovskite composition. Exploring novel interfacial additives or materials to alter the interface strain without causing phase or structural variability between the perovskite and charge transport layers. 3) Building a collaborative strategy for defect passivation is important, using the multidimensional collaborative from process control, component design, interface engineering, and external strain regulation combined with solvent engineering, antisolvent strategy, component engineering, and additive intervention to achieve the strain release. 4) Starting from the crystal nucleation and growth process, using the advanced testing technology combined with first‐principles analysis, tracing back to the source, comprehensively and thoroughly expounding the strain release and stability mechanism of perovskite and PSCs.

The pursuit of highly effective and stable PSCs toward ultimate commercialization necessitates a greater knowledge of the sources, effects, and regulators of strain in light of the significance of strain for PSCs.

## Conflict of Interest

The authors declare no conflict of interest.
